# A hierarchical Bayesian inference model for volatile multivariate exponentially distributed signals

**DOI:** 10.3389/fncom.2025.1408836

**Published:** 2025-11-12

**Authors:** Changbo Zhu, Ke Zhou, Fengzhen Tang, Yandong Tang, Xiaoli Li, Bailu Si

**Affiliations:** 1 State Key Laboratory of Robotics, Shenyang Institute of Automation, Chinese Academy of Sciences, Shenyang, China; 2 Institutes for Robotics and Intelligent Manufacturing, Chinese Academy of Sciences, Shenyang, China; 3 University of Chinese Academy of Sciences, Beijing, China; 4 Beijing Key Laboratory of Applied Experimental Psychology, School of Psychology, Beijing Normal University, Beijing, China; 5 State Key Laboratory of Cognitive Neuroscience and Learning, Beijing Normal University, Beijing, China; 6 School of Systems Science, Beijing Normal University, Beijing, China; 7 Chinese Institute for Brain Research, Beijing, China

**Keywords:** online Bayesian learning, hierarchical filter, Brownian motion, exponential distribution, adaptive observation

## Abstract

Brain activities often follow an exponential family of distributions. The exponential distribution is the maximum entropy distribution of continuous random variables in the presence of a mean. The memoryless and peakless properties of an exponential distribution impose difficulties for data analysis methods. To estimate the rate parameter of multivariate exponential distribution from a time series of sensory inputs (i.e., observations), we constructed a hierarchical Bayesian inference model based on a variant of general hierarchical Brownian filter (GHBF). To account for the complex interactions among multivariate exponential random variables, the model estimates the second-order interaction of the rate intensity parameter in logarithmic space. Using variational Bayesian scheme, a family of closed-form and analytical update equations are introduced. These update equations also constitute a complete predictive coding framework. The simulation study shows that our model has the ability to evaluate the time-varying rate parameters and the underlying correlation structure of volatile multivariate exponentially distributed signals. The proposed hierarchical Bayesian inference model is of practical utility in analyzing high-dimensional neural activities.

## 1 Introduction

Decoding of the states of neural systems is a critical task for many applications in neural engineering, ranging from cognitive assessment, brain–machine interface to deep brain stimulation (Haynes and Rees, 2006; Qi et al., 2019; Yousefi et al., 2019; Xu et al., 2021; Zhang et al., 2022; Pan et al., 2022; Li and Le, 2017). However, there are several critical challenges faced by mental state decoding methods. First, brain activities are highly non-stationary, often showing transient dynamics. Second, responses of different brain regions are correlated, due to the dense complex anatomical connectivity patterns. Third, imaging processes of brain activities imposed additional spatial temporal transformations on neural signals, calling for appropriate inference methods to uncover the underlying brain states. To tackle these difficulties, methods that are capable of tacking and inferring multi-dimensional dynamic brain signals are indispensable.

Brain activities are shown to follow particular types of distributions that are distinctive from Gaussian distributions (Roxin et al., 2011). Extracellular recordings of brain voltage signals of various brain regions from different animals could be described by an exponential family of distributions, with tails falling off according to exponential distributions (Swindale et al., 2021). The distributions of the electromyography and electroencephalography signals from human subjects are found to have fatter tails than that of a Gaussian distribution and are fitted well by a generalized extreme value distribution (Nazmi et al., 2015). The innate statistics of the measured neural activities lead the direct application of classic tracking and inference methods, such as Kalman filtering, to be suboptimal (Li et al., 2009; Malik et al., 2010). It is therefore a valuable research direction to develop inference methods that closely match the characteristics of brain activities.

Exponential distributions well describe empirical data in neuroscience. Neurons in many regions, such as middle temporal and medial superior temporal visual areas in monkeys, fire in a Poisson-like fashion, with exponential distributed interspike intervals (Maimon and Assad, 2009; Ouyang et al., 2023). The sleep episode durations of human and other mammals, such as cats and rats, follow exponential distributions (Lo et al., 2004). The locomotion activity of cells in vitro displays a universal exponential distribution (Czirók et al., 1998). In addition, exponential distribution provides a good description of waiting times in the physical world, including lifespans, counts within a finite time period and so on. Therefore, researchers employ exponential distribution as lifetime distribution model to describe the lifetimes of manufactured products (Davis, 1952; Epstein and Sobel, 1953; Varde, 1969) and the survival or remission times in chronic diseases (Shanker et al., 2015). In physics, an exponential distribution is the best model of the times between successive flaps of a flag for a variety of wind speeds (McCaslin and Broussard, 2007). In finance, accumulating evidences have suggested that financial data can be quantified by exponential distributions. A study of tax and census data shows an exponential distribution of individual income in the United States (Drăgulescu and Yakovenko, 2001). An exponential distribution also agrees well with income for families with two earners (Drăgulescu and Yakovenko, 2001).

In this article, we aim to develop an inference model particularly to deal with the problem of volatility and multi-dimensionality in data space. Importantly, we assume that the data follow a multivariate exponential distribution, capturing the fat tail characteristics of neural signals. The proposed model can be applied to state estimation tasks in psychophysics, brain activity analysis, as well as other non-linear time series modeling tasks.

In probability theory, exponential distribution is a maximum entropy distribution of a continuous random variable with a bounded mean (Jaynes, 1982; Conrad, 2004; Stein et al., 2015). The exponential distribution has several interesting and important properties (Johnson et al., 2002; Ibe, 2014; Marshall and Olkin, 1967b):

- An exponential distribution is governed by a rate parameter (interpreted as the inverse of average waiting time). The mean of an exponential random variable is equal to the standard deviation (std).
- Exponential distribution is peakless. The probability density function of an exponential distribution is monotonously decreasing. The expectation of an exponential random variable is not at the maximum point of its probability density function. This means that samples drawn from an exponential distribution contain high noise, resulting in a fat tail.
- An exponential random variable is memoryless, i.e.,

$$\begin{array}{l} P\left(x>t+\epsilon |x>\epsilon \right)=P\left(x>t\right),\forall t,\epsilon >0. \end{array}$$

In a Poisson process, this memoryless property means that the probability of waiting time until the next event is not affected by start time (Kingman, 1992). All waiting times are independently identically distribution (iid).

Due to these characteristics, fitting models of multivariate exponential distribution is a difficult problem encountered in various disciplines. The Marshall–Olkin exponential distribution is introduced based on shock models and the constraint that residual life and age are independent (Marshall and Olkin, 1967a). An exponential distribution with exponential minimums provides a model to describe the reliability of a coherent system (Esary and Marshall, 1974). A bivariate generalized exponential distribution is also introduced to analyze lifetime data in two dimensions (Kundu and Gupta, 2009). However, these models are complex in form and are not robust for non-stationary data. More importantly, the interactions among the components of a multivariate exponential variable are not trivial to estimate. These classical studies took the assumption of static distributions, without considering the dynamic changes of the underlying distributions. Robust methods for the estimation of multivariate exponential distribution in volatile environments are still sparse.

“Observing the observer” is a meta Bayesian framework (Daunizeau et al., 2010b,a) and furnishes a unified programming and modeling framework that unites perception and action based on the variational free energy principle (Beal, 2003; Friston, 2010; Mathys et al., 2011; Friston et al., 2017). Perceptual and response models are two major parts of this framework. Inversion of the perceptual and response models can map from sensory inputs (i.e., observations) into response actions. Following this framework, the general hierarchical Brownian filter (GHBF) was proposed as a model for state estimate in dynamic multi-dimensional environments with Gaussian distribution assumption (Zhu et al., 2025). An important function of this model is to capture temporal dynamics of lower order interactions among sensory inputs (i.e., observations).

In this article, we extend the general hierarchical Brownian filter to non-Gaussian case and develop an inference model for volatile multivariate exponentially distributed signals. The inference model incorporates a hierarchical perceptual model and a response model into the “observing the observer” framework. The model receives a series of multidimensional sensory inputs or observations and is asked to infer rate parameter of a multivariate exponential distribution in a complex volatile environment. The perceptual model represents rate parameter and covariance of the logarithm of rate parameter. The response model is a stochastic mapping to reproduce a series of sensory inputs. Compared with previous hierarchical Bayesian methods (Beal, 2003; Friston, 2010; Mathys et al., 2011; Friston et al., 2017), the proposed model is able to deal with multidimensional signals and dynamically uncover the potential correlation structure in the data.

The contribution of this article is two-fold. First, we develop a hierarchical Bayesian model to estimate the parameters of multivariate exponential distributions which are subject to dynamic changes. Through variational Bayesian learning, the model infers the rate parameters and the pairwise correlations of multivariate exponentially distributed signals at the same time; therefore, it is able to robustly track the distribution dynamically. The proposed model is valuable for its potential applications in estimating neural and behavioral responses. Second, the efficiency and the robustness of the proposed inference model is tested in simulations with synthetic dynamic data. Compared with a simplified model of constant volatility parameters, the proposed model is better in explaining the data, demonstrating the importance role of higher order variables, such as correlations, in estimating the parameters of the signal.

The rest of this article is structured as follows. The mathematical notations used in this study is defined in Section 2. Section 3 introduces the hierarchical Bayesian perceptual model in multivariate exponential distribution environment. Section 4 derives a set of closed form update equations for perceptual inference. Simulations results are given in Section 6. Finally, the article is concluded after discussions.

## 2 Notations

Throughout this article, we use the following conventional mathematical notations:

- A bold capital letter is a matrix while a bold lowercase letter is a vector.
- A hollow capital letter denotes a set, which is also denoted by {}.
- A probability density function (PDF) is denoted by *q*(·) or *p*(·).
- A multivariate Gaussian PDF of *x* is denoted by N(x;μ,Σ) with mean μ and variance Σ, while a multivariate Gaussian random vector is denoted by x~N(μ,Σ).
- An multivariate exponential PDF of *x* can be denoted by E(x;r) with a rate parameter *r*, while an multivariate exponential random vector is denoted by x~E(r).
- A sequence of variables over time are denoted by “:,” for example,

$$o_{1:K}=o\left(t_{1}\right),o\left(t_{2}\right),\cdots \;,o\left(t_{K}\right).$$

- *E*_*q*(*x*)_(*v*) means the expectation of *v* under the distribution *q*(*x*).
- The operator ⊙ is the Hadamard product, the operation diag(*v*) is to transform a vector *v* into a diagonal square matrix with the elements of *v* on the principal diagonal.
- The function vec(*M*_*m*×*n*_) is the vectorization of a matrix *M*, a linear operation, to obtain a column vector of length *m*×*n* by concatenating the columns of the matrix *M* consecutively from column 1 to column *n*. The operator ⊗ is the Kronecker product.
- The function lvec(*L*) is to transform a lower triangular matrix *L* into a column vector lvec(*L*) obtained by stacking columns without zero elements in the upper triangle part of the matrix.

## 3 Hierarchical Bayesian perceptual model

### 3.1 Parameterization of multivariate exponential distribution

Given a random multivariate exponential variable *x*_0_ without cross dimension interactions among components, we can easily get the joint probability of all components by directly multiplying all marginal exponential distributions:


$$\begin{array}{l} E\left(x_{0};r_{0}\right)=\overset{d_{0}}{\underset{i=1}{\prod }}r_{0}^{\left(i\right)}\exp\left(-r_{0}^{\left(i\right)}x_{0}^{\left(i\right)}\right)=\exp\left(-r_{0}^{T}x_{0}\right)\overset{d_{0}}{\underset{i=1}{\prod }}r_{0}^{\left(i\right)}, \end{array}$$ (1)

where $x_{0}^{\left(i\right)}$ is the *i*-th component (i.e., random exponential variable) of *x*_0_. The rate parameter $r_{0}^{\left(i\right)}$ is the expectation of the *i*-th random exponential variable $x_{0}^{\left(i\right)}$. *r*_0_ is the expected rate vector of random vector *x*_0_. The integer *d*_0_ is the number of dimensions of the random vector *x*_0_. However, this independent model is incapable of capturing the pairwise probabilistic correlation among the components of *x*_0_. If we introduce non-independent exponential model with interactions among the components of *x*_0_, it will lead to high model complexity. Since the rate parameter *r*_0_ is of primary interest, we aim to learn the rate parameter by explicitly considering the pairwise interactions among the components of *r*_0_. To keep the positive constraint of the rate parameter, we convert the constrained learning problem into an unconstrained learning in logarithmic space. More specifically, the rate *r*_0_ is mapped from a point *x*_1_ in its log-space


$$\begin{array}{l} r_{0}\left(t\right)=\exp\left(W_{1}x_{1}\left(t\right)+b_{1}\right), \end{array}$$ (2)

where the notation exp(·) denotes the element-wise exponential function. The coefficient matrix *W*_1_ is a diagonal matrix with positive elements on the principal diagonal. This matrix represents the coupling strength between *x*_0_ and *x*_1_. The bias *b*_1_ is a shift parameter.

### 3.2 Perceiving tendency and volatility of the rate parameter

**Figure 1:**
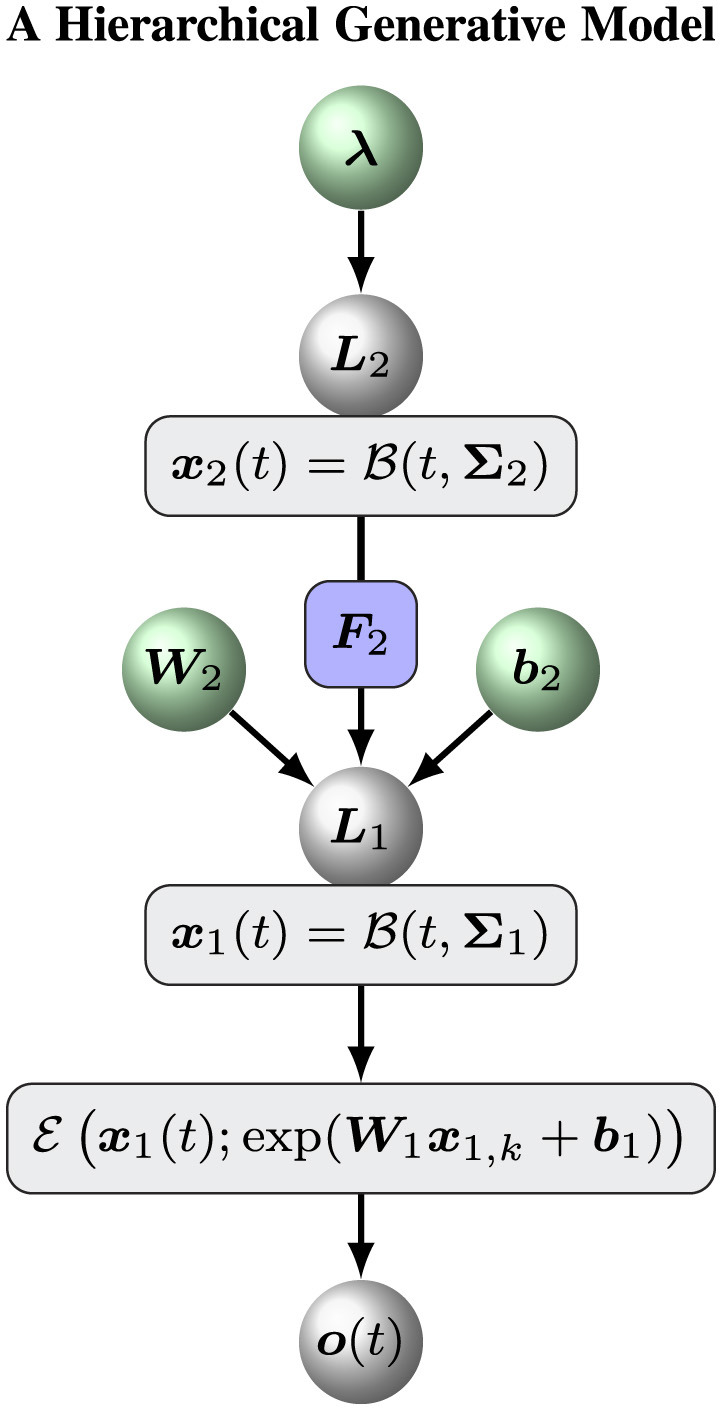
Overview of the hierarchical perceptual model.

Volatile signals fluctuate over time, showing variations. The fluctuations of the signals are again subject to changes, and so forth. The nested nature of volatility is a hallmark of collective phenomena as observed in many complex systems like brain network, animal swarm and financial market. To quantitatively describe volatility and pairwise correlations of multi-dimensional signals, general hierarchical volatility model could be constructed based on nested Brownian motions (Zhu et al., 2025). The basic idea is that the variable of interest is represented by a Brownian motion, while the changes of the variable is predicted by higher order variables that are again subject to Brownian motions. Following this framework, we develop a hierarchical perceptual model to estimate the tendency and volatility of multivariate exponentially distributed signals (Figure 1). More specifically, the logarithms of rate parameters *x*_1_ of the underlying multivariate exponential distribution is modeled by a general Brownian motion with diffusion matrix $\Sigma_{1}\in {\mathbb{R}}^{d_{1}\times d_{1}}$


$$\begin{array}{l} x_{1}=B\left(t;\Sigma_{1}\right). \end{array}$$ (3)

This Brownian motion captures the tendency of the learned parameter vector *x*_1_. The volatility (i.e., uncertainties and pairwise correlations) in *x*_1_ is given by $\Sigma_{1}\in {\mathbb{R}}^{d_{1}\times d_{1}}$, which is a symmetric positive definite matrix by definition. Considering the fact that the diffusion matrix Σ_1_ is a symmetric positive definite matrix, it could be uniquely represented by a lower triangular matrix $L_{1}\in {\mathbb{R}}^{d_{1}\times d_{1}}$ according to Cholesky decomposition (Tanabe and Sagae, 1992; Jung and O'Leary, 2006):


$$\begin{array}{l} \Sigma_{1}=L_{1}{L_{1}}^{T}. \end{array}$$

To further evaluate the volatility Σ_1_ in *x*_1_, we assume that its decomposition *L*_1_ is modeled by a general Brownian motion in its parameterized space. To be exact, the elements of *L*_1_ is parametrized by a *d*_2_ = *d*_1_(*d*_1_+1)/2 dimensional vector *y*_2_, which results from concatenating the lower triangle elements of *L*_1_ in a column-wise fashion. The element in *i*-th row and *j*-th column of *L*_1_ is parameterized by


$$\begin{array}{l} {L_{1}}^{\left(i,j\right)}=l_{1}^{\left(i,j\right)}=\\ \{\begin{array}{ll} 2\sinh\left(y_{2}^{\left(\frac{\left(2d_{1}-j+2\right)\left(j-1\right)}{2}+i-j+1\right)}\right), & 1\leq j<i\leq d_{1}\\ \exp\left(y_{2}^{\left(\frac{\left(2d_{1}-i+2\right)\left(i-1\right)}{2}+1\right)}\right), & j=i \end{array} \end{array}$$ (4)

where sinh(·) denotes the hyperbolic sine function. Note that Equation 4 transforms *L*_1_ into logarithmic space, while conserving non-negativity for diagonal elements and allowing arbitrary values for off-diagonal elopements of *L*_1_.

The vector *y*_2_ represents the volatility of the signal in logarithmic space, therefore constitutes a parameterization of the volatility. *y*_2_ is given by the following mapping in the second level of the model:


$$\begin{array}{l} y_{2}=W_{2}x_{2}+b_{2}, \end{array}$$ (5)

where *b*_2_ and $x_{2}\in {\mathbb{R}}^{d_{2}}$ represent the trend and time-varying fluctuation in log-volatility of *x*_1_, respectively. The coefficient matrix *W*_2_ is a *d*_2_-by-*d*_2_ diagonal matrix representing the coupling strength from level two to level one. *W*_2_ can simply take the form of a diagonal matrix spanned from a column vector *w*_2_ with all positive elements


$$\begin{array}{l} {W_{2}}^{\left(i,i\right)}=w_{2}^{\left(i\right)}. \end{array}$$

We can rewrite the coupling (Equations 4, 5) as


$$\begin{array}{l} L_{1}=F_{2}\left(x_{2};w_{2},b_{2}\right). \end{array}$$

In the second level of the model, we further assume that *x*_2_ evolves as a general Brownian motion with diffusion matrix $\Sigma_{2}\in {\mathbb{R}}^{d_{2}\times d_{2}}$


$$\begin{array}{l} x_{2}=B\left(t;\Sigma_{2}\right). \end{array}$$ (6)

The diffusion matrix Σ_2_ is chosen as a diagonal matrix for simplicity. Let $L_{2}\in {\mathbb{R}}^{d_{2}\times d_{2}}$ be the unique Cholesky decomposition of Σ_2_. We simply assume that *L*_2_ is a constant diagonal matrix spanned by vector $\lambda \in {\mathbb{R}}^{d_{2}}$ with all elements being positive.

Figure 1 shows an overview of the hierarchical perceptual model. With this model, a Bayesian agent receives a series of sensory inputs or observations *o*_1:*T*_. At time *t*_*k*_, the sensory input *o*_*k*_ to the agent is determined by a delta distribution δ(·)


$$\begin{array}{l} P\left(o_{k}|x_{0,k}\right)=\delta \left(o_{k}=x_{0,k}\right). \end{array}$$ (7)

The initial priori states *p*(*x*_1, 0_, *x*_2, 0_) are Gaussian distributions as follows:


$$\begin{array}{l} \begin{array}{cc} q\left(x_{h,0}\right)= & N\left(x_{h,0};\mu_{h,0},C_{h,0}\right),h=1,2. \end{array} \end{array}$$ (8)

In summary, the hierarchical perceptual model constitutes a generative model for sensory observations *o*(*t*) based on hidden representations of the tendency (*x*_1_) and the volatility (*x*_2_) of the observations. . To simplify the notations, we introduced the notation *X* to denote the set of all hidden states, ℙ for the hyperparameters and the prior states of the model:


$$\begin{array}{l} X=\left\{{\text{x}}_{0},{\text{x}}_{1},{\text{x}}_{2}\right\},\\ \mathbb{P}=\left\{{\text{w}}_{1},{\text{b}}_{1},{\text{w}}_{2},{\text{b}}_{2},\lambda ,\mu_{1,0},{\text{C}}_{1,0},\mu_{2,0},{\text{C}}_{2,0}\right\} \end{array}$$

where μ_1, 0_, *C*_1, 0_, μ_2, 0_, and*C*_2, 0_ are the prior states of the model defined in Equation 8 and Supplementary material Section 2.

## 4 Perceptual inference approximated by variational approximation

The aforementioned hierarchical perceptual model is constructed based on general continuous Brownian motions. It remains to derive update rules to estimate the posterior distributions for the hidden representations *x*_1_ and *x*_2_. In order to derive a family of analytical and efficient update rules, we discretize continuous Brownian motions by applying the Eulerian method. The sampling interval (SI) ϵ_*k*_ = *t*_*k*_−*t*_*k*−1_ is defined by the time that elapses between the arrival of consecutive sensory inputs *o*_*k*−1_ and *o*_*k*_.

We use the variational Bayesian method (Beal, 2003; Friston, 2010; Daunizeau et al., 2010b; Mathys et al., 2011) to reach an approximation to the posterior distributions of *x*_1_(*t*) and *x*_2_(*t*) given the sensory input *o*(*t*) (i.e., observation). To this end, we maximize the negative free energy, which is the lower bound of log-model evidence, to yield variational approximation posterior (cf. Supplementary material Section 1):


$$\begin{array}{l} q\left(x_{h,k}\right)=\frac{1}{Z_{h}}\exp\left(V_{h}\left(x_{h,k}\right)\right),h=1,2, \end{array}$$ (9)

where $Z_{h}$ is a normalization constant. *V*_*h*_(*x*_*h, k*_) is the variational energy given by


$$\begin{array}{l} V_{h}\left(x_{h,k}\right)=E_{q\left(X_{\backslash h,k}\right)}\left[\ln\;p\left(X_{k},o_{k}|\mathbb{P},\epsilon_{k}\right)\right]. \end{array}$$ (10)

Here we introduced the notation *X*_\*h, k*_ for excluding *x*_*h, k*_ from the set *X*_*k*_, Then under Brownian and Gaussian assumptions, the approximation variational posterior (Zhu et al., 2025) is


$$\begin{array}{l} {\text{x}}_{h,k}|{\text{o}}_{k},\mathbb{P}\sim N(\mu_{h,k},{\text{C}}_{h,k}),\\ h=1,2. \end{array}$$ (11)

Under this approximation, the inference of the posterior distributions of *x*_*h*_ is reduced to the estimation of the mean μ_*h, k*_ and the covariance matrix *C*_*h, k*_, or equivalently the precision matrix $P_{h,k}\equiv {\left(C_{h,k}\right)}^{-1}$. Following (Zhu et al., 2025), the update rules for the posterior distributions of *x*_1_ and *x*_2_ are derived.

At the bottom (zeroth) level of the hierarchical perceptual model, we can directly determine multivariate exponential distribution *q*(*x*_0, *k*_) with the expectation:


$$\begin{array}{l} {\text{μ}}_{0,k}={\text{o}}_{k}. \end{array}$$ (12)

At the first level, following Equation 10, *V*_1_(*x*_1_) is calculated as


$$\begin{array}{l} \;V_{1}({\text{x}}_{1,k})=E_{q(X_{\backslash 2,k})}[\ln p(X_{k},{\text{o}}_{k}|\mathbb{P},\epsilon_{k})]\\ =\ln p({\text{o}}_{k}|{\text{x}}_{0,k})+E_{q({\text{x}}_{0,k})}[\ln p({\text{x}}_{0,k}|{\text{x}}_{1,k})]\\ \;+E_{q({\text{x}}_{2,k})}[\ln p({\text{x}}_{1,k}|{\text{x}}_{2,k},{\text{W}}_{2},{\text{b}}_{2},\epsilon_{k})]\\ \approx 1^{T}({\text{W}}_{1}{\text{x}}_{1,k}+{\text{b}}_{1})-\mu_{0,k}^{T}\text{exp}\left({\text{W}}_{1}{\text{x}}_{1,k}+{\text{b}}_{1}\right)\\ \;-\frac{1}{2}({\text{x}}_{1,k}-{\text{μ}}_{1,k-1}){)}^{T}(\epsilon_{k}{\hat{\Sigma }}_{1,k}+C_{1,k-1}{)}^{-1}({\text{x}}_{1,k}-{\text{μ}}_{1,k-1})\\ \;+\text{const} \end{array}$$ (13)

where 1 is a *d*_0_ dimensional column vector in which all elements are 1. Here we use the approximation


$$\begin{array}{l} {\left(\epsilon_{k}\Sigma_{1,k}+C_{1,k-1}\right)}^{-1}\approx {\left(\epsilon_{k}{\hat{\Sigma }}_{1,k}+C_{1,k-1}\right)}^{-1}, \end{array}$$ (14)

with ${\hat{\Sigma }}_{1,k}$ computed from the second level


$$\begin{array}{l} {\hat{\Sigma }}_{1,k}={\hat{L}}_{1,k}{\hat{L}}_{1,k}^{T},\\ {\hat{L}}_{1,k}=F_{2}(\mu_{2,k-1};w_{2},b_{2}). \end{array}$$ (15)

The variational energy *V*_1_(*x*_1, *k*_) is not a standard Gaussian quadratic form, so we have to employ a Gaussian quadratic form to approximate it (Zhu et al., 2025). To obtain this approximation form, we give the gradient and Hessian matrix of *V*_1_(*x*_1, *k*_) as follows:


$$\begin{array}{l} \nabla V_{1}(x_{1,k})=W_{1}^{T}\left[1-\mu_{0,k}⊙\exp\left(W_{1}x_{1,k}+b_{1}\right)\right]\\ \;-(\epsilon_{k}{\hat{\Sigma }}_{1,k}+C_{1,k-1}{)}^{-1}(x_{1,k}-\mu_{1,k-1}), \end{array}$$ (16)

and


$$\begin{array}{l} \nabla^{2}V_{1}(x_{1,k})=-W_{1}^{T}\text{diag}\left(\mu_{0,k}⊙\exp\left(W_{1}x_{1,k}+b_{1}\right)\right)W_{1}\\ \;-(\epsilon_{k}{\hat{\Sigma }}_{1,k}+C_{1,k-1}{)}^{-1}, \end{array}$$ (17)

Under the Gaussian quadratic form approximation, which is based on a single step Newton method (Zhu et al., 2025), the tendency of *x*_0, *k*_ is captured by


$$\begin{array}{l} \mu_{1,k}=\mu_{1,k-1}+C_{1,k}W_{1}^{T}{PE}_{0,k}, \end{array}$$ (18)

where *PE*_0, *k*_ is the prediction error:


$$\begin{array}{l} {PE}_{0,k}=1-\mu_{0,k}⊙{\hat{r}}_{0,k}. \end{array}$$ (19)

${\hat{r}}_{0,k}\equiv {\left[{\hat{r}}_{0,k}^{\left(1\right)},{\hat{r}}_{0,k}^{\left(2\right)},\cdots \;,{\hat{r}}_{0,k}^{\left(d_{0}\right)}\right]}^{T}$ is the prediction given by the mapping in Equation 2:


$$\begin{array}{l} {\hat{r}}_{0,k}=\exp\left(W_{1}\mu_{1,k-1}+b_{1}\right). \end{array}$$ (20)

Unpacking prediction error *PE*_0, *k*_ results in a meaningful formula,


$$\begin{array}{l} PE_{0,k}^{\left(i\right)}=1-\mu_{0,k}^{\left(i\right)}{\hat{r}}_{0,k}^{\left(i\right)}=1-\frac{\mu_{0,k}^{\left(i\right)}}{\frac{1}{{\hat{r}}_{0,k}^{\left(i\right)}}}. \end{array}$$

The inverse of the predicted rate $\frac{1}{{\hat{r}}_{0,k}^{\left(i\right)}}$ gives the expectation of sensory input, and the ratio $\frac{\mu_{0,k}^{\left(i\right)}}{\frac{1}{{\hat{r}}_{0,k}^{\left(i\right)}}}$ measures the accuracy of the prediction. If the ratio is greater than 1 (i.e., the predicted expectation of sensory input is less than the actual sensory input), the prediction error is negative, and the agent should decrease $\mu_{1}^{\left(i\right)}$. If the ratio is less than 1, the prediction error is positive, the agent should increase $\mu_{1}^{\left(i\right)}$, so that the predicted expectation of sensory input could be decreased. Ideally, the ratio is equal to 1,and the prediction error vanishes, which means that the predicted expectation of the sensory input is equal to the actual sensory input.

In Equation 18, the prediction error is scaled and rotated by the covariance matrix *C*_1, *k*_ of the approximate Gaussian distribution, which is converted from the precision matrix:


$$\begin{array}{l} \begin{array}{l} C_{1,k}\equiv {\left(P_{1,k}\right)}^{-1},\\ {\;P}_{1,k}={\hat{\Pi }}_{1,k}+W_{1}^{T}\mathrm{diag}\left(\mu_{0,k}⊙{\hat{r}}_{0,k}\right)W_{1}. \end{array} \end{array}$$ (21)

Here prediction precision ${\hat{\Pi }}_{1,k}$ is given by


$$\begin{array}{l} {\hat{\Pi }}_{1,k}={\left(\epsilon_{k}{\hat{\Sigma }}_{1,k}+C_{1,k-1}\right)}^{-1}. \end{array}$$ (22)

Note that the off-diagonal elements of the inverse prediction precision matrix ${\hat{\Pi }}_{1,k}$ give the prediction correlations.

At the second level, the volatility, consisting of the uncertainties and pairwise correlations in natural parameters, is inferred by similar variational approximation method (Zhu et al., 2025). The mean is updated by


$$\begin{array}{l} \begin{array}{cc} \mu_{2,k} & =\mu_{2,k-1}+\epsilon_{k}C_{2,k}W_{2}^{T}{\hat{L}}_{g1,k}\left({\hat{\Omega }}_{1,k}⊗I_{d_{1}}\right)\text{vec}\left(\Delta_{1,k}^{T}\right). \end{array} \end{array}$$ (23)

Here Δ_1, *k*_ is given by


$$\begin{array}{ll} \Delta_{1,k}= & \left[C_{1,k}+{PE}_{1,k}{PE}_{1,k}^{T}\right]{\hat{\Pi }}_{1,k}-I_{d_{1}}. \end{array}$$ (24)

The constant matrix *I*_*d*_1__ is a *d*_1_-by-*d*_1_ unit square matrix. *PE*_1, *k*_ is the prediction error on the hidden state *x*_1_


$$\begin{array}{l} {PE}_{1,k}=\mu_{1,k}-\mu_{1,k-1}. \end{array}$$ (25)

${\hat{L}}_{g1,k}$ is given by


$$\begin{array}{l} \begin{array}{c} {\hat{L}}_{g1,k}=\left[\begin{array}{c} \exp\left({\left(W_{2}^{\left(1\right)}\right)}^{T}\mu_{2,k-1}+b_{2}^{\left(1\right)}\right)e_{2}^{T}\left(1\right)\\ 2\cosh\left({\left(W_{2}^{\left(2\right)}\right)}^{T}\mu_{2,k-1}+b_{2}^{\left(2\right)}\right)e_{2}^{T}\left(2\right)\\ \exp\left({\left(W_{2}^{\left(3\right)}\right)}^{T}\mu_{2,k-1}+b_{2}^{\left(3\right)}\right)e_{2}^{T}\left(3\right)\\ 2\cosh\left({\left(W_{2}^{\left(4\right)}\right)}^{T}\mu_{2,k-1}+b_{2}^{\left(4\right)}\right)e_{2}^{T}\left(4\right)\\ \vdots \\ \exp\left({\left(W_{2}^{\left(d_{2}\right)}\right)}^{T}\mu_{2,k-1}+b_{2}^{\left(d_{2}\right)}\right)e_{2}^{T}\left(d_{2}\right) \end{array}\right] \end{array}, \end{array}$$ (26)

where the constant vector *e*_2_(*d*_2_) is a $d_{1}^{2}$-dimension column vector. The *j*-th component in $e_{2}^{T}\left(d_{2}\right)$ is 1 if *j* = *i* or 0 if *j*≠*i*. The column vector $W_{2}^{\left(i\right)}$ is the *i*-th row in the coefficient matrix *W*_2_. ${\hat{\Omega }}_{1,k}$ is defined as


$$\begin{array}{l} {\hat{\Omega }}_{1,k}={\hat{L}}_{1,k}^{T}{\hat{\Pi }}_{1,k}. \end{array}$$ (27)

The precision matrix is updated by


$$\begin{array}{l} P_{2,k}={\hat{\Pi }}_{2,k}+W_{2}^{T}{\hat{L}}_{g1,k}\{\epsilon_{k}^{2}K_{d_{1}d_{1}}\\ \;\left[\;{\hat{\Omega }}_{1,k}^{T}⊗[{\hat{\Omega }}_{1,k}\Delta_{1,k}]+[\Delta_{1,k}^{T}{\hat{\Omega }}_{1,k}^{T}]⊗{\hat{\Omega }}_{1,k}+{\hat{\Omega }}_{1,k}^{T}⊗{\hat{\Omega }}_{1,k}\right]\\ \;+\epsilon_{k}^{2}[\left[{\hat{L}}_{1,k}^{T}\Delta_{1,k}^{T}{\hat{\Omega }}_{1,k}^{T}]⊗{\hat{\Pi }}_{1,k}+[{\hat{L}}_{1,k}^{T}{\hat{\Omega }}_{1,k}^{T}]⊗[{\hat{\Pi }}_{1,k}\Delta_{1,k}\right]\\ \;+[{\hat{L}}_{1,k}^{T}{\hat{\Omega }}_{1,k}^{T}]⊗{\hat{\Pi }}_{1,k}\;\;]-\epsilon_{k}\left[I_{d_{1}}⊗[{\hat{\Pi }}_{1,k}\Delta_{1,k}]\right]\}\\ \;{\hat{L}}_{g1,k}^{T}W_{2}-W_{2}^{T}\text{diag}\left(\text{lvec}\left(\delta_{1,k}\right)\right)W_{2}, \end{array}$$ (28)

where


$$\delta_{1,k}=\epsilon_{k}\left[\Delta_{1,k}^{T}{\hat{\Omega }}_{1,k}^{T}\right]⊙{\hat{L}}_{1,k}$$

The precision matrix of the prediction ${\hat{\Pi }}_{2}$ is given by


$$\begin{array}{l} {\hat{\Pi }}_{2,k}={\left(\epsilon_{k}\Sigma_{2}+C_{2,k-1}\right)}^{-1}. \end{array}$$ (29)

The notation *K*_*mn*_ denotes a *mn*-by-*mn* commutation matrix (Magnus and Neudecker, 1979).

## 5 Variational Bayesian learning

A model M with a set of parameters ℙ receives and encodes sensory input *o*(*t*). We can arrange all elements of ℙ into a vector ξ. Here, we introduce the following mean field approximation to fit the parameters of the model with the sensory inputs *o*_1:*K*_


$$\begin{array}{l} \begin{array}{l} q\left(\mathbb{P}\right)=q\left(\xi \right)=q\left(w_{1}\right)q\left(b_{1}\right)q\left(w_{2}\right)q\left(b_{2}\right)q\left(\lambda \right)\\ \;\cdot q\left(\mu_{1,0}\right)q\left(C_{1,0}\right)q\left(\mu_{2,0}\right)q\left(C_{2,0}\right). \end{array} \end{array}$$ (30)

Then


$$\begin{array}{l} \begin{array}{l} \ln\;p\left(o_{1:K}|M\right)=\ln\;\int p\left(o_{1:K},\xi |M\right)d\xi \\ =\ln\;\int \frac{p\left(o_{1:K},\xi |M\right)q\left(\xi \right)}{q\left(\xi \right)}d\xi \\ \geq \int q\left(\xi \right)\ln\;\left(\frac{p\left(o_{1:K},\xi |M\right)}{q\left(\xi \right)}\right)d\xi \\ =\int q\left(\xi \right)\ln\;p\left(o_{1:K},\xi |M\right)-q\left(\xi \right)\ln\;q\left(\xi \right)d\xi \\ ≜F_{M}\left(\xi \right) \end{array}. \end{array}$$ (31)

We use the Lagrange multiplier method to work out the optimal variational posterior as follows:


$$\begin{array}{l} \begin{array}{l} q\left(\xi \right)=\frac{1}{Z_{\xi }}\exp\left(V\left(\xi \right)\right)\\ V\left(\xi \right)=\ln\;p\left(o_{1:K},\xi |M\right). \end{array} \end{array}$$ (32)

Then we execute a Laplacian approximation to determine a Gaussian approximation of the variational posterior solution (Equation 33)


$$\begin{array}{l} \begin{array}{l} \mu_{\xi }=\underset{\xi }{\mathrm{argmax}}V\left(\xi \right)=\underset{\xi }{\mathrm{argmax}}\ln\;p\left(o_{1:K},\xi |M\right)\\ \;=\underset{\xi }{\mathrm{argmax}}\ln\;p\left(o_{1:K}|\xi ,M\right)p\left(\xi \right)\\ \;=\underset{\xi }{\mathrm{argmax}}\overset{K}{\underset{k=1}{\sum }}\ln\;p\left(o_{k}|\xi ,M\right)+ln\;p\left(\xi \right)\\ \;=\underset{\xi }{\mathrm{argmax}}\overset{K}{\underset{k=1}{\sum }}\ln\;p\left(o_{k}|{\hat{r}}_{0,k},\xi ,M\right)+ln\;p\left(\xi \right),\\ \;C_{\xi }=-\frac{\partial^{2}V\left(\mu_{\xi }\right)}{\partial \xi \partial \xi^{T}}, \end{array} \end{array}$$ (33)

where $\ln\;p\left(o_{k}|\xi ,M\right)$ is the logarithm of the predictive distribution $o_{k}\sim E\left({\hat{r}}_{0,k}\right)$ and is given by


$$\begin{array}{l} \ln\;p\left(o_{k}|{\hat{r}}_{0,k},\xi ,M\right)=1^{T}\ln\;{\hat{r}}_{0,k}-o_{k}^{T}{\hat{r}}_{0,k}. \end{array}$$ (34)

Finally, the maximum value $F_{M}\left(\mu_{\xi },C_{\xi }\right)$ of the negative free energy $F_{M}\left(\xi \right)$ is given by


$$\begin{array}{l} \begin{array}{c} F_{M}\left(\xi \right)\leq F_{M}\left(\mu_{\xi },C_{\xi }\right)=V\left(\mu_{\xi }\right)+\frac{d_{\xi }}{2}\ln\;2\pi e+\frac{1}{2}\ln\;\det\left(C_{\xi }\right). \end{array} \end{array}$$ (35)

## 6 Simulation study

To verify the effectiveness of the proposed model, we conducted simulations on synthetic data to assess the model's ability to capture time-varying rate parameters of multivariate exponential distribution. The purpose of using simulation is to validate the model on precisely defined data, so that the results given by the model could be compared with ground truth.

### 6.1 An ablation model

**Figure 2:**
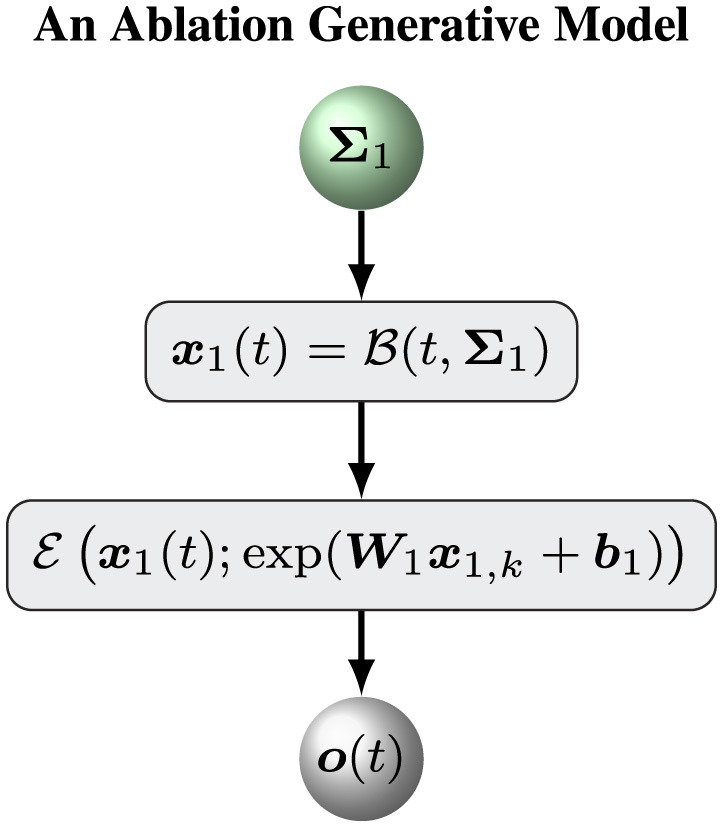
Overview of the ablation model.

To assess the ability of our hierarchical Bayesian model M, we define an ablation model $M_{a}$ as a baseline model to evaluate the role of the top (volatility) level of the hierarchical Bayesian model M. Put simply, an ablation model $M_{a}$ is the simple version of the hierarchical Bayesian model M with a constant volatility *x*_2_(*t*) = μ_2_. In this case, we can remove the variable *x*_2, *k*_ and keep a constant likelihood matrix Σ_1_. The model $M_{a}$ can be defined by Equations 1–3. Figure 2 shows the overall framework of the ablation model $M_{a}$.

The update equations for the ablation model are similar to Equations 12, 18–22 with ${\hat{\Sigma }}_{1}=\Sigma_{1}$. Put simply, we assume that Σ_1_ is a diagonal matrix with positive diagonal elements. Therefore, Σ_1_ can be determined by a vector σ_1_ with positive elements. The prior distribution of Σ_1_ is defined by


$$\begin{array}{l} q\left(\Sigma_{1}\right)=q\left(\ln\;\sigma_{1}\right)=N\left(\ln\;\sigma_{1};\mu_{\ln\;\sigma_{1}},C_{\ln\;\sigma_{1}}\right) \end{array}$$ (36)

where μ_ln_σ__1__, *C*_ln_σ__1__ are the parameters of the prior distribution. Other parameters of this model are the same prior model with the above hierarchical Bayesian model (cf. Supplementary material Section 2).

### 6.2 Simulation setup

**Figure 3:**
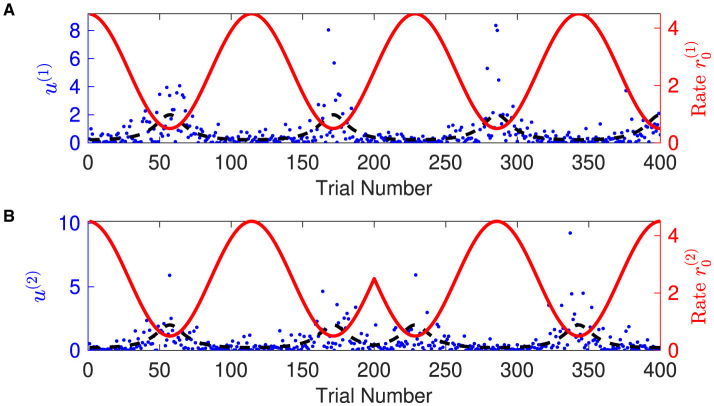
Time-varying rate parameter and sensory inputs of volatile multivariate exponentially distributed signals. Panels **(A, B)** represent two dimensions of input signal. In each panel, blue dots are the sensory inputs *o*^(*i*)^ of the *i*-th dimension of the signal. Red lines represent the expected rate $r_{0}^{\left(i\right)}\left(t\right)$. The black dashed lines are the expectation of the sensory input *o*^(*i*)^, i.e., the inverse of the expected rate $r_{0}^{\left(i\right)}\left(t\right)$. Note that the expected rate in the two dimensions fluctuates in time, synchronously before and anti-synchronously after trial 200.

In detail, simulations were carried out in four steps as follows:

1. Generating synthetic sensory inputs. We randomly generated a sequence of bivariate exponential variable *o*_1:*K*_ = *o*(*t*_1_), *o*(*t*_2_), *o*(*t*_3_), ⋯ , *o*(*t*_*K*_) (*K* = 400) (Figure 3):

$$\begin{array}{l} p\left(o\left(t\right)\right)=E\left(o\left(t\right),r_{0}\left(t\right)\right), \end{array}$$ (37)

where the time-varying rate vector *r*_0_(*t*) was governed by cosine waves and was defined by


$$\begin{array}{l} \begin{array}{l} r_{0}^{\left(1\right)}\left(t_{k}\right)=2.5+2\cos\left(\frac{7\pi }{K}t_{k}\right),\\ r_{0}^{\left(2\right)}\left(t_{k}\right)=\{\begin{array}{l} 2.5+2\cos\left(\frac{7\pi }{K}t_{k}\right)k\leq 200\\ 2.5-2\cos\left(\frac{7\pi }{K}t_{k}\right)k\geq 201 \end{array}. \end{array} \end{array}$$

2. Initializing the sufficient statistics of all random parameters. We must choose particular initial sufficient statistics of a parameter vector ξ (Table 1 for the hierarchical Bayesian model and Table 2 for the ablation model) to make the models work well on a sequence of sensory inputs. Then we determined the prior distribution of ξ. All parameter configurations for the two models (Figures 1, 2) are shown in Tables 1, 2.
3. Maximizing negative free energy. We employed optimization methods to obtain the optimal sufficient statistics (μ_ξ_, *C*_ξ_) of the prior parameter ξ. The quasi-Newton Broyden-Fletcher-Goldfarb-Shanno method based on a line search framework (Nocedal and Wright, 2006) was adopted to maximize negative free energy (Equations 31, 33, 34) (Beal, 2003; Friston, 2010).
4. Generating the optimal trajectories of all states. We use the optimal prior parameters μ_ξ_ to characterize a particular model (Figures 1, 2). The two models are compared on inference and decision-making tasks.

**Table 1:** Parameters of the hierarchical Bayesian model.

**Name**	**Description**	**Initial value**	**Fixed or free**
**Parameters of the hierarchical Bayesian model**			
*d*_*o*_	Dimension of *o*	2	Constant
*d*_1_	Dimension of *x*_1_	2	Constant
*d*_2_	Dimension of *x*_2_	3	Constant
ϵ_*k*_	Sampling interval ϵ_*k*_	1	Constant
α_λ_	Upper bound on λ	0.04·1	constant
λ	Volatility of *x*_2_		Fixed
$\mu_{\lambda^{G}}$	Mean of λ^*G*^	0	
$C_{\lambda^{G}}$	Covariance of λ^*G*^	*I*_*d*_2__	
α_*w*_2__	Upper bound on *w*_2_	1	constant
*w*_2_	Coupling strength		Fixed
$\mu_{w_{2}^{G}}$	Mean of $w_{2}^{G}$	0	
$C_{w_{2}^{G}}$	Covariance of $w_{2}^{G}$	*I*_*d*_2__	
*b*_2_	Coupling bias	0	Fixed
μ_*b*_2__	Mean of *b*_2_	0	
*C*_*b*_2__	Covariance of *b*_2_	*O*_3_	
μ_2, 0_	Prior mean of *x*_2_		Free
μ_μ_2, 0__	Mean of μ_2, 0_	[0, 0, 0]^*T*^	
*C*_μ_2, 0__	Covariance of μ_2, 0_	$1\times 10^{-1}\cdot I_{3}$	
*C*_2, 0_	Prior covariance of *x*_2_		Fixed
$\mu_{c_{2}^{G}}$	Mean of $c_{2}^{G}$	[0, 0, 0]^*T*^	
$C_{c_{2}^{G}}$	Covariance of $c_{2}^{G}$	*I*_*d*_2__	
μ_1, 0_	Prior mean of *x*_1_		Free
μ_μ_1, 0__	Mean of μ_1, 0_	[25, 25]^*T*^	
*C*_μ_1, 0__	Covariance of μ_1, 0_	*I*_*d*_1__	
*C*_1, 0_	Prior covariance of *x*_1_		Free
$\mu_{c_{1}^{G}}$	Mean of $c_{1}^{G}$	0	
$C_{c_{1}^{G}}$	Covariance of $c_{1}^{G}$	*I*_*d*_1__	
*w*_1_	Coupling strength		Fixed
$\mu_{w_{1}^{G}}$	Mean of $w_{1}^{G}$	[ln(0.06), ln(0.06)]^*T*^	
$C_{w_{2}^{G}}$	Covariance of $w_{1}^{G}$	*O*_*d*_1__	
*b*_1_	Coupling bias	0	Fixed
μ_*b*_1__	Mean of *b*_1_	0	
*C*_*b*_1__	Covariance of *b*_1_	*O*_*d*_1__	

All parameters of the proposed hierarchical Bayesian model are listed in the table. Parameters labeled by “Free” are optimized by the inversion of the model. Fixed parameters are constant and were not optimized. The notation 1 is a constant column vector in which all components are 1. 0 is a zero vector. The bold letter *O*_*d*_ represents a *d* by *d* constant matrix in which all elements are 0. Given all the initial priors, we can search the optimal priors on all optimized parameters μ_ξ_ according to free energy principle (Equations 31, 33).

**Table 2:** Parameters of the ablation model.

**Name**	**Description**	**Initial value**	**Fixed or free**
**Parameters of the ablation model**			
*d*_*o*_	Dimension of *o*	2	Constant
*d*_1_	Dimension of *x*_1_	2	Constant
ϵ_*k*_	Sampling interval ϵ_*k*_	1	Constant
σ_1_	Volatility of *x*_1_		Fixed
$\mu_{\sigma_{1}^{G}}$	Mean of $\sigma_{1}^{G}$	[ln 0.01, ln 0.01]^*T*^	
$C_{\sigma_{1}^{G}}$	Covariance of $\sigma_{1}^{G}$	*I*_*d*_1__	
μ_1, 0_	Prior mean of *x*_1_		Free
μ_μ_1, 0__	Mean of μ_1, 0_	[25, 25]^*T*^	
*C*_μ_1, 0__	Covariance of μ_1, 0_	*I*_*d*_1__	
*C*_1, 0_	Prior covariance of *x*_1_		Free
$\mu_{c_{1}^{G}}$	Mean of $c_{1}^{G}$	[ln 0.25, ln 0.25]^*T*^	
$C_{c_{1}^{G}}$	Covariance of $c_{1}^{G}$	*I*_*d*_1__	
*w*_1_	Coupling strength		Fixed
$\mu_{w_{1}^{G}}$	Mean of $w_{1}^{G}$	[ln(0.06), ln(0.06)]^*T*^	
$C_{w_{2}^{G}}$	Covariance of $w_{1}^{G}$	*O*_*d*_1__	
*b*_1_	Coupling bias	0	Fixed
μ_*b*_1__	Mean of *b*_1_	0	
*C*_*b*_1__	Covariance of *b*_1_	*O*_*d*_1__	

All parameters of the ablation model are listed in the table. Parameters labeled by “Free” are optimized by the inversion of the model. Fixed parameters are constant and were not optimized. The notation 1 is a constant column vector in which all components are 1. 0 is a zero vector. The bold letter *O*_*d*_ represents a *d* by *d* constant matrix in which all elements are 0. Given all the initial priors, we can search the optimal priors on all optimized parameters μ_ξ_ according to free energy principle (Equations 31, 33).

### 6.3 Perceiving volatile multivariate exponentially distributed signals

The proposed hierarchical Bayesian inference model endowed with the optimal parameter μ_ξ_ constitutes a hierarchical Bayesian agent. We asked the hierarchical Bayesian agent to perceive volatile multivariate exponentially distributed signals as shown in Figure 3.

**Figure 4:**
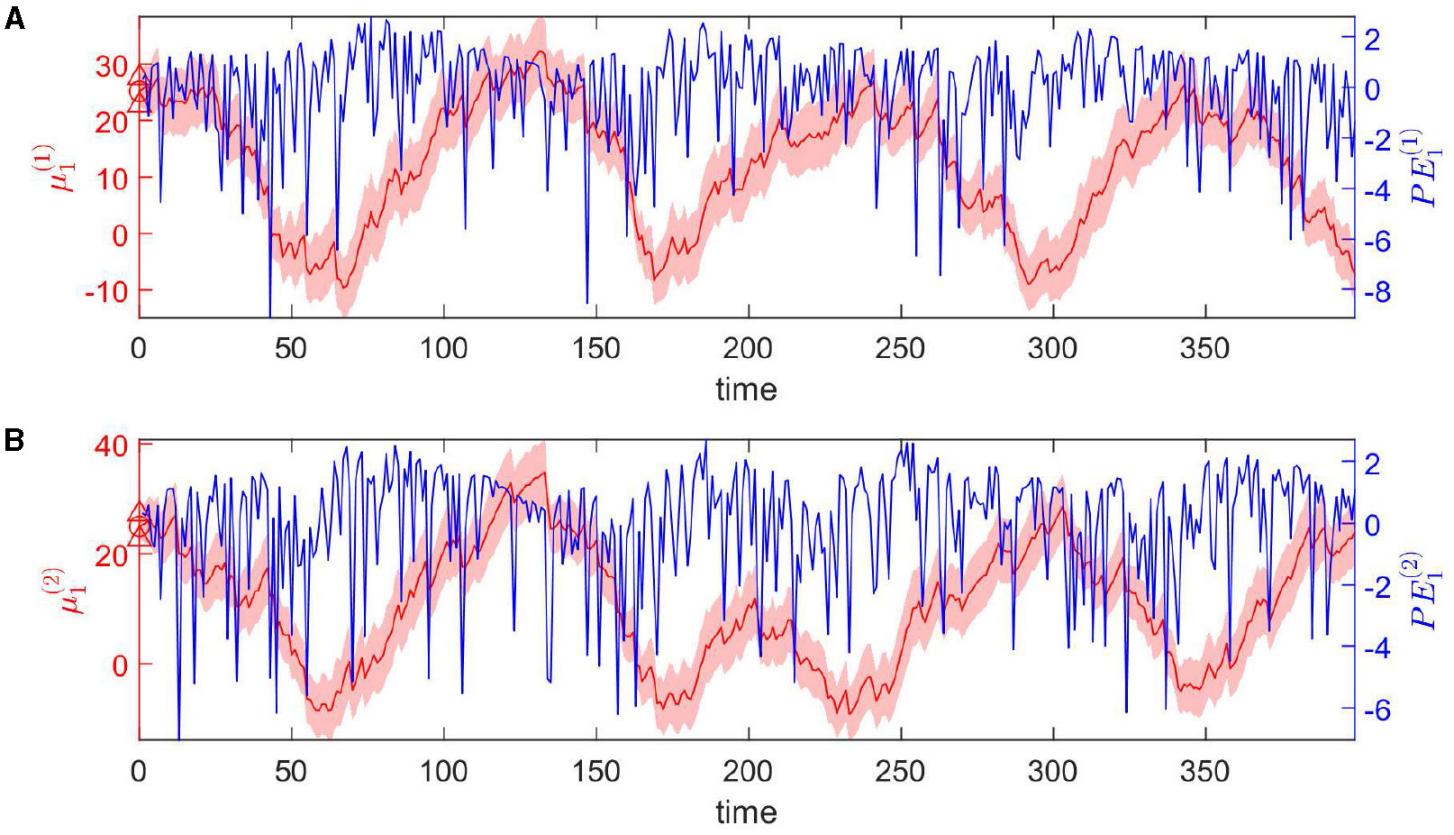
Temporal dynamics of the tendency μ_1_ of the log-rate vector *x*_1_(*t*) at the first level. Panels **(A, B)** represent two dimensions of the expectation μ_1_. In details, each panel shows one component of μ_1_ in red, and *PE*_1_ in blue. The light-red shaded area represents the uncertainty of each component (i.e., $\mu_{1}^{\left(i\right)}\left(t\right)\pm \sqrt{C_{1}^{\left(i,i\right)}\left(t\right)},i\in \left\{1,2\right\}$). The red markers △, ° represent the priors on the standard deviation and the mean of each component respectively.

The dynamic tendency μ_1_(*t*) of the log-rate vector *x*_1_(*t*) is tracked online by the hierarchical Bayesian agent (Figure 4). μ_1_ follows the varying trend of the expected rate in logarithmic space. The uncertainty of μ_1_(*t*) is stable (light-red shaded area in Figure 4). The prediction error *PE*_1_ fluctuates around a baseline (blue line in Figure 4).

**Figure 5:**
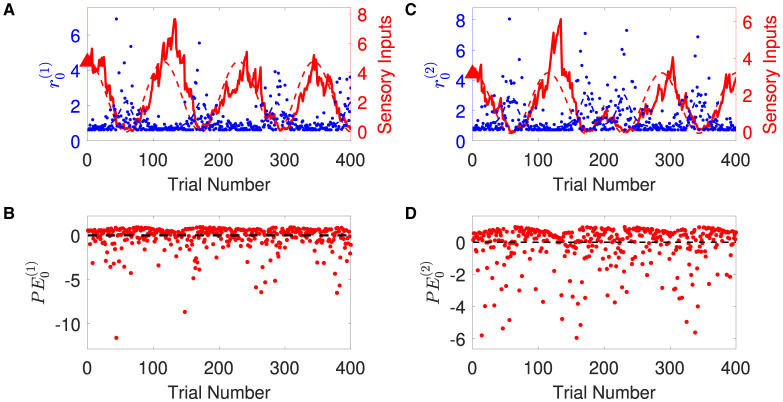
Temporal dynamics of the expectation of the logarithm of volatility μ_2_ in the state *x*_1_ at the second level. Panels **(A–C)** represent three dimensions of the expectation μ_2_. Each panel shows the evolution of one element of μ_2_ in red and the corresponding element of *PE*_2_ in blue. Light-red shaded area represents the uncertainty of each dimension (i.e., $\mu_{2}^{\left(i\right)}\left(t\right)\pm \sqrt{C_{2}^{\left(i,i\right)}\left(t\right)},i\in \left\{1,2,3\right\}$). The red markers △, ° represent the priors of the standard deviation and mean of each dimension.

Overall, the agent perceives the expected rate vector well (Figure 5). For a majority of the trials, both of the belief expectations $\mu_{0}^{\left(1\right)},\mu_{0}^{\left(2\right)}$ (solid lines in Figures 5A, C) fluctuates around the expected rate (dashed lines in Figures 5A, C). In the initial stage, the agent quickly adjusts itself to adapt to the input signal and tracks the expected states. Due to the stochasticity, the sample rate intensity in sensory inputs deviates from the expected rate intensity, leading to the estimated belief rate intensity $\mu_{0}^{\left(i\right)},i=0,1$ to deviate from the expected rate intensity. From trial 120 to trial 165, the sample rate intensity in sensory inputs *o*^(1)^ is larger than the expected rate intensity in Figure 3A. The agent's belief is higher than the expected rate (Figure 5A). From trial 116 to trial 158 (trial 296 to trial 308), the sample rate intensity in sensory inputs *o*^(2)^ is greater than the expected rate intensity in Figure 3B, leading the agent to have higher belief of the rate intensity than the expected rate value.

**Figure 6:**
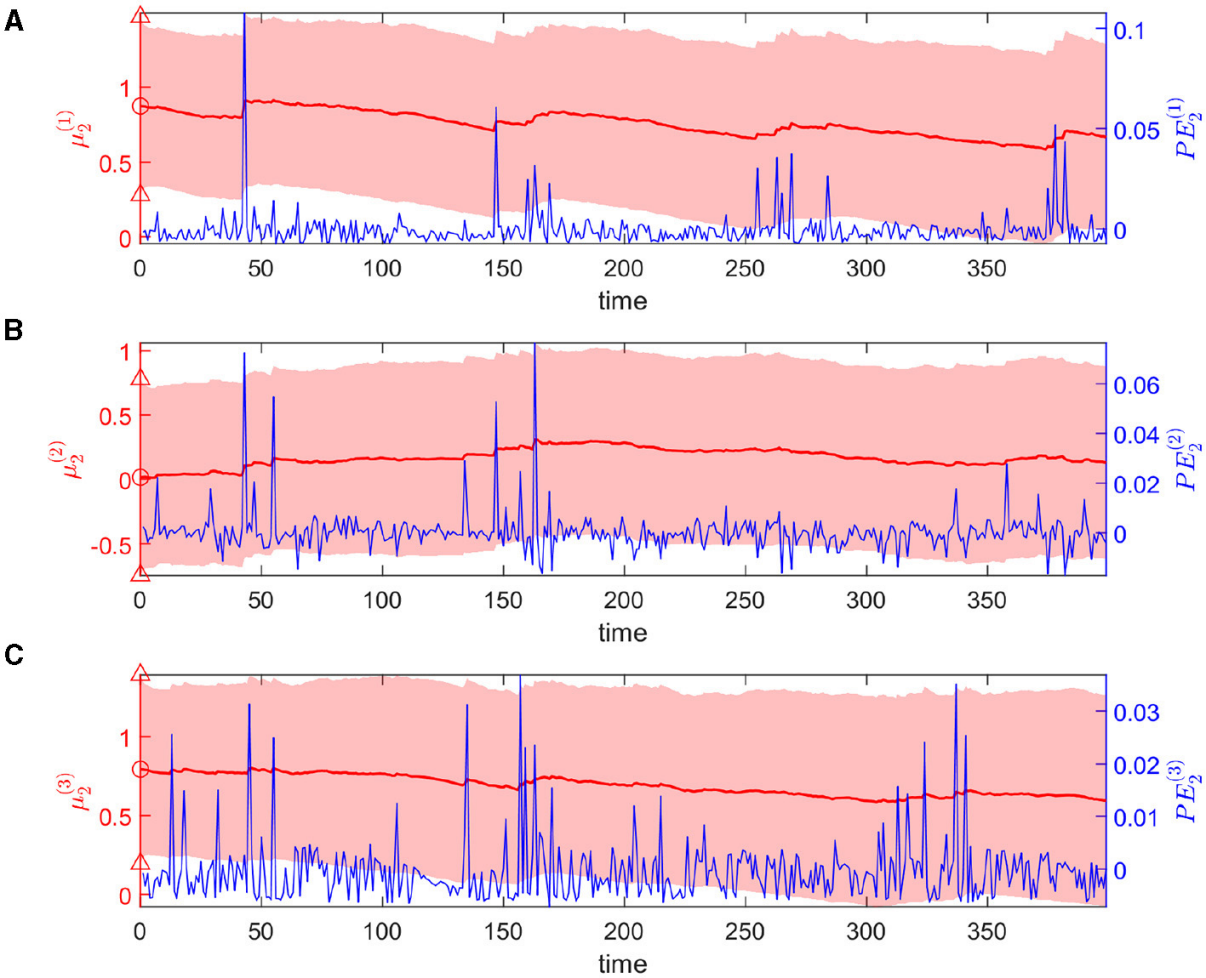
Temporal dynamics of the expectation of the logarithm of volatility μ_2_ in the state *x*_1_ at the second level. Each panel shows the evolution of one element of μ_2_ in red and the corresponding element of *PE*_2_ in blue. Light-red shaded area represents the uncertainty of each dimension (i.e., $\mu_{2}^{\left(i\right)}\left(t\right)\pm \sqrt{C_{2}^{\left(i,i\right)}\left(t\right)},i\in \left\{1,2,3\right\}$). The red markers △, ° represent the priors of the standard deviation and mean of each dimension.

**Figure 7:**
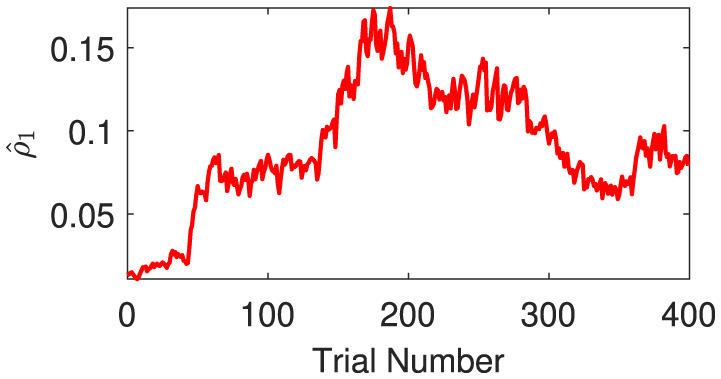
Prediction correlation ${\hat{\rho }}_{1}\left(t\right)$ is extracted from the inverse prediction precision ${\hat{\Pi }}_{1}\left(t\right)$ generated by the second (log-volatility) level.

The expectations of log-volatilities in the logarithms of the rate vector ($\mu_{2}^{\left(1\right)}$ and $\mu_{2}^{\left(2\right)}$, i.e., internal representation of the expected states) has notable changes, stabilized for most of the time (Figure 6). From trial 1 to trial 200, changes in rate $r_{0}^{\left(1\right)}$ are consistent with changes in rate $r_{0}^{\left(2\right)}$ (Figure 3). In theory, they are positively correlated during this period. From trial 1 to trial 186, the prediction correlation ${\hat{\rho }}_{1}$ continues to increase (Figure 7). From trial 187 to trial 200, asynchronous local fluctuations (or noise) lead to a decrease in prediction correlation ${\hat{\rho }}_{1}$. From trial 201 to trial 400, changes in rate $r_{0}^{\left(1\right)}$ are the opposite with the changes in rate $r_{0}^{\left(2\right)}$ (Figure 3). The two dimensions of the signal are negatively correlated during this period. As a result, the prediction correlation ${\hat{\rho }}_{1}$ of the agent continues to decrease from trial 201 to trial 359. From trial 359 to trial 365, prediction errors $PE_{1}^{\left(1\right)}\text{and}PE_{1}^{\left(2\right)}$ are positive numbers, and drive prediction correlation ${\hat{\rho }}_{1}$ to jump to a larger value (Figure 7). The hierarchical Bayesian agent therefore is able to uncover the correlation structures of the signal dynamically.

### 6.4 Bayesian model selection

**Figure 8:**
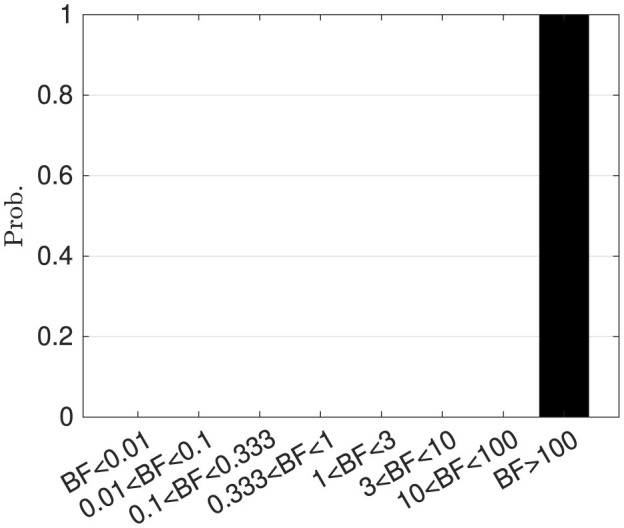
Histogram of Bayesian factors. Bayesian factor with the Bayesian information criterion $BF\left(M,M_{a}\right)$.

To compare the performance of the proposed hierarchical Bayesian model M and the ablation model $M_{a}$, we performed 100 independent simulations for each model using different seeds of random number generators. Based on these simulations, Bayesian factors were calculated. Figure 8 shows the histogram of the Bayesian factors $BF\left(M,M_{a}\right)$. According to the criteria suggested by Harold Jeffreys (cf. Supplementary material Section 4), M is better than $M_{a}$.

## 7 Discussion

### 7.1 Contributions of this study

In this article, we developed a hierarchical Bayesian model to infer and track online the tendency and volatility in multivariate exponential signals. The bottom level of the hierarchical Bayesian model is to learn the expected rate parameter vector of the multivariate exponential signal. The logarithm of the rate parameter vector *x*_1_ is modeled to evolve as a general Brownian motion at the first level. Under the Brownian and Gaussian assumption on *x*_1_, the volatility in *x*_1_ can be computed by the Cholesky decomposition of the diffusion matrix of the Brownian motion *x*_1_. Therefore, we introduce a parameterization of the volatility in *x*_1_ in logarithmic space after the Cholesky decomposition of the diffusion matrix of *x*_1_. The volatility in *x*_1_ can be represented by *x*_2_, which again evolves as a Brownian motion. The low-order interactions among the components of the log-rate parameter vector and uncertainties are captured by *x*_2_ at the second level of the model.

The hierarchical Bayesian model assumes that the log-rate parameter vector *x*_1_(*t*) evolves as a general Brownian motion and can be updated by Equation 18, where prediction error *PE*_0, *k*_ drives the agent to diminish the difference between the agent's belief and the sensory input. The coefficient matrix *W*_1_ plays the role of scaling factors to weight prediction error *PE*_0, *k*_. The covariance *C*_1, *k*_ functions as complex adaptive learning rate in Equation 21.

In principle, the proposed model could be easily generalized to a Bayesian framework for decision making in high-dimensional volatile environments by defining appropriate form of response models (Berger, 2013; Mathys et al., 2014; Zhu et al., 2022). In this article, we define a simple random response model based on bivariate exponential distribution. For other problems of interest, it is sufficient to construct a compatible response model addressing the particular optimization criteria of the question.

### 7.2 Limitations and strengths

The peakless and memoryless properties of the exponential distribution bring difficulties for an online agent to predict, since historical sensory inputs can only provide weak evidence for a prediction. The proposed hierarchical Bayesian agent internally integrates historical sensory inputs and the current sensory input to infer the changes in the signal. The agent estimates the dynamic volatility in the sensory inputs and adjusts the learning rate based on the evidence of the volatility, so that the information from the signal is integrated into the internal states efficiently. The proposed hierarchical Bayesian agent is able to efficiently and accurately capture the characteristics of volatile multivariate exponentially distributed signals.

In the simulation, we observed that the proposed hierarchical Bayesian agent has good suppression effect on small volatility, but it is also swayed by the local variation of the rate intensity caused by the stochasticity of the signal. The prediction correlation is not only determined by changes in the trend of the sensory inputs but is also affected by volatility. Large local fluctuations can also cause jumps in prediction correlations. Asynchronous persistent small local fluctuations will also reduce the prediction correlation, while synchronous persistent small fluctuations will increase the prediction correlation.

In this study, we simply considered simulated data, which aims to capture dynamic and multidimensional aspects of nonstationary multivariate exponential signals and cannot cover other important features observed in real data set. The results obtained from simulations pave ways for further investigations of many estimation problems in neuroscience research. The possible applications of the method include firing rate estimation, functional brain connection estimation, etc.

## 8 Conclusions

We have introduced the mathematical basis of a hierarchical Bayesian model for inferring and tracking rate intensity parameter of multivariate exponential signals and illustrated its functionality. A family of interpretable closed form update rules were derived. In particular, we provided a full theoretical scenario that consists of inference in the perceptual model and learning optimal hyper-parameters by inversion of the hierarchical Bayesian model. The proposed theoretical framework was validated on synthetic data, and it turned out that the hierarchical Bayesian model worked well in tracking volatile multi-variate exponential signals. The preliminary study here points to the practical utility of our approach in analyzing high-dimensional neural activities, which often follow as distributions in exponential family.

## Data Availability

The original contributions presented in the study are included in the article/Supplementary material, further inquiries can be directed to the corresponding author.
