# Supplementary material for: A hierarchical Bayesian inference model for volatile multivariate exponentially distributed signals
Source: Front Comput Neurosci. 2025 Nov 12;19:1408836. doi: 10.3389/fncom.2025.1408836 (PMC12648510; doi:10.3389/fncom.2025.1408836)
Supplement: Supplementary file 1 [file Presentation_1.pdf]

Supplementary Material

1 VARIATIONAL BAYESIAN INFERENCE

Given a Bayesian perceptual model $p(\mathbb{X}, \boldsymbol{o}|\mathbb{P}, \epsilon)$, where $\mathbb{P}$ and ϵ are parameters, the model evidence $p(\boldsymbol{o}|\mathbb{P}, \epsilon)$ is often analytically intractable. Therefore, exact Bayesian posteriors could not be analytically calculated. We apply variational Bayesian methods to transform the calculation of exact Bayesian posteriors $p(\mathbb{X}|\boldsymbol{o}, \mathbb{P}, \epsilon)$ into finding the optimal variational posteriors $q(\mathbb{X})$. The lower bound on the logarithm of the model evidence $p(\boldsymbol{o}|\mathbb{P}, \epsilon)$ is given by

$$\begin{aligned}
\ln p(\boldsymbol{o}|\mathbb{P}, \epsilon) &= \ln \int q(\mathbb{X}) \frac{p(\boldsymbol{o}, \mathbb{X}|\mathbb{P}, \epsilon)}{q(\mathbb{X})} \mathrm{d}\mathbb{X} \\
&\geq \int q(\mathbb{X}) \ln \frac{p(\boldsymbol{o}, \mathbb{X}|\mathbb{P}, \epsilon)}{q(\mathbb{X})} \mathrm{d}\mathbb{X} \\
&= \int q(\mathbb{X}) \ln p(\boldsymbol{o}, \mathbb{X}|\mathbb{P}, \epsilon) \mathrm{d}\mathbb{X} - \int q(\mathbb{X}) \ln q(\mathbb{X}) \mathrm{d}\mathbb{X} \\
&= - U(\mathbb{X}) + H(\mathbb{X}) \\
&= \mathcal{F}(q(\mathbb{X})).
\end{aligned} \tag{S1}$$

Then we use an important assumption that marginal variational posteriors over latent variables are independent, i.e. the joint variational posterior distribution factorizes with respect to all marginal posteriors

$$q(\mathbb{X}) = \prod_{\boldsymbol{x}_h \in \mathbb{X}} q(\boldsymbol{x}_h), \tag{S2}$$

where $\boldsymbol{x}_h$ is one element of $\mathbb{X}$. The factorized form in Equation S2 corresponds to the so-called *mean field approximation*, an approximation scheme developed in statistical mechanics.

It should be noted that we now wish to maximize the negative free energy $\mathcal{F}(q(\mathbb{X}))$ with respect to each approximation posterior $q(\boldsymbol{x}_h)$ under the constraint of normalized probability $\int q(\boldsymbol{x}_h) \mathrm{d}\boldsymbol{x}_h = 1, \forall h$. The

Lagrange functional $\bar{F}(q(\mathbb{X}))$ is defined as

$$\begin{aligned}
\bar{\mathcal{F}}(q(\mathbb{X})) =& \bar{\mathcal{F}}(q(\mathbb{X}_{\backslash h}), q(\boldsymbol{x}_h)) \\
\triangleq& \mathcal{F}(q(\mathbb{X})) + \sum_{h=1}^{H} \kappa_h \left(\int q(\boldsymbol{x}_h) d\boldsymbol{x}_h - 1 \right) \\
=& - \int q(\mathbb{X}) \ln p(\mathbb{X}, \boldsymbol{o}|\mathbb{P}, \epsilon) \mathrm{d}\mathbb{X} + \int q(\mathbb{X}) \ln q(\mathbb{X}) \mathrm{d}\mathbb{X} \\
& + \sum_{h=1}^{H} \kappa_h \left(\int q(\boldsymbol{x}_h) \mathrm{d}\boldsymbol{x}_h - 1 \right) \\
=& - \int q(\mathbb{X}_{\backslash h}) q(\boldsymbol{x}_h) \ln p(\mathbb{X}, \boldsymbol{o}|\mathbb{P}, \epsilon) \mathrm{d}\mathbb{X}_{\backslash h} \mathrm{d}\boldsymbol{x}_h \\
& + \int q(\mathbb{X}_{\backslash h}) q(\boldsymbol{x}_h) \ln q(\mathbb{X} \backslash \{\boldsymbol{x}_h\}) q(\boldsymbol{x}_h) d\mathbb{X}_{\backslash h} \mathrm{d}\boldsymbol{x}_h \\
& + \sum_{i \in \mathbb{H}_{\backslash h}} \kappa_i \left(\int q(\boldsymbol{x}_i) \mathrm{d}\boldsymbol{x}_i - 1 \right) + \kappa_h \left(\int q(\boldsymbol{x}_h) \mathrm{d}\boldsymbol{x}_h - 1 \right),
\end{aligned} \tag{S3}$$

where κ_h is a Lagrange Multiplier. We use $\mathbb{X}_{\backslash h}$ to denote the set defined by the subtraction of two sets $\mathbb{X} - \{\boldsymbol{x}_h\}$, the notation $\mathbb{H}$ for an index set $\{1, 2, 3, ..., H\}$, and the notation $\mathbb{H}_{\backslash h}$ for the subtraction of two sets $\mathbb{H} - \{h\}$. The variation of Equation S3 with respect to $q(\boldsymbol{x}_h)$ is

$$\begin{aligned}
\frac{\delta \bar{\mathcal{F}}(q(\mathbb{X}))}{\delta q(\boldsymbol{x}_h)} =& \frac{\delta \bar{\mathcal{F}}(q(\mathbb{X}_{\backslash h}), q(\boldsymbol{x}_h))}{\delta q(\boldsymbol{x}_h)} \\
=& - \int q(\mathbb{X}_{\backslash h}) \ln p(\mathbb{X}, \boldsymbol{o}|\mathbb{P}, \epsilon) \mathrm{d}\mathbb{X}_{\backslash h} \\
& + \int q(\mathbb{X}_{\backslash h}) \ln q(\mathbb{X}_{\backslash h}) \mathrm{d}\mathbb{X}_{\backslash h} \\
& + \ln q(\boldsymbol{x}_h) + \kappa_h + 1 \\
=& 0.
\end{aligned} \tag{S4}$$

The optimal variational approximation posterior $q(\boldsymbol{x}_h)$ is of the form of Boltzmann distribution

$$\begin{aligned}
q(\boldsymbol{x}_h) =& \frac{1}{\mathcal{Z}_h} \exp\left(V_h(\boldsymbol{x}_h)\right) \\
V_h(\boldsymbol{x}_h) =& \int q(\mathbb{X}_{\backslash h}) \ln p(\mathbb{X}, \boldsymbol{o}|\mathbb{P}, \epsilon) d\mathbb{X}_{\backslash h} \\
H_e(\mathbb{X}_{\backslash h}) =& - \int q(\mathbb{X}_{\backslash h}) \ln q(\mathbb{X}_{\backslash h}) \mathrm{d}\mathbb{X}_{\backslash h} \\
\mathcal{Z}_h =& \exp(-H_e(\mathbb{X}_{\backslash h}) + \kappa_h + 1),
\end{aligned} \tag{S5}$$

where $V_h(\boldsymbol{x}_h) = \int q(\mathbb{X}_{\backslash h}) \ln p(\mathbb{X}, \boldsymbol{o}|\mathbb{P}, \epsilon) d\mathbb{X}_{\backslash h}$ corresponds to negative internal energy over the hidden variable $\boldsymbol{x}_h$. The quantity $V_h(\boldsymbol{x}_h)$ is often called variational energy.

2 PRIOR DISTRIBUTIONS OF PARAMETERS

In a Bayesian model, an unknown parameter can be treated as a random variable. Probability models could be employed to determine the parameters. Put simply, the probability density function of each random parameter is modeled by a delta-function at each time, and their values follow various multivariate Gaussian distributions (Beal, 2003; Mathys et al., 2011). In addition, different parameters may have different constraints, therefore we introduce parameterizations to represent these constrained parameters (Zhu et al., 2025).

In the coupling between the bottom level and the first level, we have introduced principle diagonal coefficient matrix $\boldsymbol{W}_1$, with all positive diagonal elements and a bias vector $\boldsymbol{b}_1$ in Equation **??**. The vector $\boldsymbol{w}_1$ denotes all free elements in $\boldsymbol{W}_1$, $\boldsymbol{w}_1 = \boldsymbol{diag}(\boldsymbol{W}_1)$. We represent these free parameters in logarithmic space to preserve nonnegativity. More specifically, $\boldsymbol{w}_1$ is expressed in its log-space by a Gaussian random vector $\boldsymbol{w}_1^G$

$$\begin{aligned}
&\boldsymbol{w}_1^{(i)} = \exp(\boldsymbol{w}_1^{(i)G}), \boldsymbol{w}_1^{(i)G} \in \mathbb{R} \\
&\boldsymbol{w}_1 = \mathbf{exp}(\boldsymbol{w}_1^G) \\
&q(\boldsymbol{w}_1) = q(\boldsymbol{w}_1^G) = \mathcal{N}(\boldsymbol{w}_1^G; \boldsymbol{\mu}_{\boldsymbol{w}_1^G}, \boldsymbol{C}_{\boldsymbol{w}_1^G}).
\end{aligned} \tag{S6}$$

We may assume that the bias $\boldsymbol{b}_1$ is a multivariate Gaussian distribution with the mean $\boldsymbol{\mu}_{\boldsymbol{b}_1}$ and the variance $\boldsymbol{C}_{\boldsymbol{b}_1}$

$$q(\boldsymbol{b}_1) = \mathcal{N}(\boldsymbol{b}_1; \boldsymbol{\mu}_{\boldsymbol{b}_1}, \boldsymbol{C}_{\boldsymbol{b}_1}). \tag{S7}$$

The coupling mapping $\boldsymbol{F}_2$ contains bias $\boldsymbol{b}_2$ and coupling strength $\boldsymbol{w}_2$ as parameters (Equation **??**). We make an assumption on $\boldsymbol{b}_2$ that it is a multivariate Gaussian distribution with the mean $\boldsymbol{\mu}_{\boldsymbol{b}_2}$ and the variance $\boldsymbol{C}_{\boldsymbol{b}_2}$

$$q(\boldsymbol{b}_2) = \mathcal{N}(\boldsymbol{b}_2; \boldsymbol{\mu}_{\boldsymbol{b}_2}, \boldsymbol{C}_{\boldsymbol{b}_2}). \tag{S8}$$

In principle, there should not be any constraints on the coupling strength $\boldsymbol{w}_2$. However, there is no reason to choose $\boldsymbol{w}_2$ to a be negative elements, since the negativity in $\boldsymbol{w}_2$ could be counterbalanced by the negativity in $\boldsymbol{x}_2$. Therefore, the lower bound on each component $\boldsymbol{w}_2^{(i)}$ is chosen to be 0. In addition, considering the fact that $\boldsymbol{w}_2$ is involved in the update of the positive definite precision matrix $\boldsymbol{P}_2$ (Equation **??**), each component $\boldsymbol{w}_2^{(i)}(i = 1, 2, ..., d_2)$ should have a upper bound. If the value of $\boldsymbol{w}_2^{(i)}$ is too large, $\boldsymbol{P}_2$ would be degenerated. To avoid such violations, we set the upper bound of $\boldsymbol{w}_2^{(i)}$ to be a constant value $\boldsymbol{\alpha}_{\boldsymbol{w}_2}^{(i)} > 0$, i.e. the i-th component of a constant column vector $\boldsymbol{\alpha}_{\boldsymbol{w}_2}$. We use a sigmoid function to map a multivariate Gaussian variable into a bounded variable $\boldsymbol{w}_2$. This transformation and the priors on $\boldsymbol{w}_2$ are given as

$$\begin{aligned}
&\boldsymbol{w}_2^{(i)} = \boldsymbol{W}_2^{(i,i)} = \frac{\boldsymbol{\alpha}_{\boldsymbol{w}_2}^{(i)}}{1 + \exp(-\boldsymbol{w}_2^{(i)G})}, \forall i \in \{1, 2, \cdots, d_2\} \\
&\boldsymbol{w}_2 = \boldsymbol{\alpha}_{\boldsymbol{w}_2} \odot \boldsymbol{s}(\boldsymbol{w}_2^G, \mathbf{1}) \\
&q(\boldsymbol{w}_2) = q(\boldsymbol{w}_2^G) = \mathcal{N}(\boldsymbol{w}_2^G; \boldsymbol{\mu}_{\boldsymbol{w}_2^G}, \boldsymbol{C}_{\boldsymbol{w}_2^G}).
\end{aligned} \tag{S9}$$

The parameter $\boldsymbol{\lambda}$ naturally has a lower bound $\mathbf{0}$ constrained by variances. But if $\boldsymbol{\lambda}$ is not bounded from above, it may cause some violations: for a large $\boldsymbol{\lambda}$, it yields small prediction precision $\hat{\boldsymbol{\Pi}}_2$ where all variances are close to 0 and causes the posterior precision $\boldsymbol{P}_2$ not to be a positive definite matrix. That is to say, an unbounded vector $\boldsymbol{\lambda}$ violates the conditions of the update equations, yielding an improbable perceptual inference. Therefore, we set an upper bound $\boldsymbol{\alpha_\lambda}$ on $\boldsymbol{\lambda}$, through a bounded sigmoid function similar as in Equation S9

$$\begin{aligned} \boldsymbol{\lambda}^{(i)} &= \frac{\boldsymbol{\alpha}_{\boldsymbol{\lambda}}^{(i)}}{1+\exp(-\boldsymbol{\lambda}^{(i)G})}, \forall i \in \{1, 2, \cdots, d_2\} \\ \boldsymbol{\lambda} &= \boldsymbol{\alpha_\lambda} \odot \boldsymbol{g}(\boldsymbol{\lambda}^G) \\ q(\boldsymbol{\lambda}) &= q(\boldsymbol{\lambda}^G) = \mathcal{N}(\boldsymbol{\lambda}^G; \boldsymbol{\mu}_{\boldsymbol{\lambda}^G}, \boldsymbol{C}_{\boldsymbol{\lambda}^G}). \end{aligned} \tag{S10}$$

where $\boldsymbol{g}(\cdot)$ is an element-wise sigmoid function.

Besides these structural parameters, the initial priors on all hidden states are also determined following similar way. In details, we use a Gaussian random variable to express the initial mean $\boldsymbol{\mu}_{h,0}$

$$\begin{aligned} & q(\boldsymbol{\mu}_{h,0}) = \mathcal{N}(\boldsymbol{\mu}_{h,0}; \boldsymbol{\mu}_{\boldsymbol{\mu}_{h,0}}, \boldsymbol{C}_{\boldsymbol{\mu}_{h,0}}) \\ & \forall h \in \{1, 2\}. \end{aligned} \tag{S11}$$

Each of the initial prior covariances $\big\{\boldsymbol{C}_{h,0} | h = 1, 2\big\}$ is restricted to a principal diagonal and positive definite matrix. All principal diagonal elements in $\boldsymbol{C}_{h,0}$ form a column vector $\boldsymbol{c_h}$. Since the components in $\boldsymbol{c_h}$ are positive, they are represented by multivariate Gaussian random variables in log-space

$$\begin{aligned} & \boldsymbol{c}_h = \mathbf{exp}(\boldsymbol{c}_h^G) \\ & q(\boldsymbol{C}_h) = q(\boldsymbol{c}_h^G) = \mathcal{N}(\boldsymbol{c}_h^G; \boldsymbol{\mu}_{\boldsymbol{c}_h^G}, \boldsymbol{C}_{\boldsymbol{c}_h^G}) \\ & \forall h \in \{1, 2\}. \end{aligned} \tag{S12}$$

3 EVALUATING NEGATIVE FREE ENERGY

There is a model $\mathcal{A}$ with parameters $\boldsymbol{\xi}$. Given a series of observations $\boldsymbol{o}_{1:K}$, we fit the model to the sensory input $\boldsymbol{o}_{1:K}$ and actions $\boldsymbol{a}_{1:K}$. There is a particular model characterized by $\boldsymbol{\xi}$. The posterior $p(\boldsymbol{\xi}|\boldsymbol{o}_{1:K}, \mathcal{A})$ on parameters $\boldsymbol{\xi}$ is approximated by a multivariate Gaussian distribution $q(\boldsymbol{\xi})$ under the *Laplacian approximation*.

$$p(\boldsymbol{\xi}|\boldsymbol{o}_{1:K}, \mathcal{A}) \approx q(\boldsymbol{\xi}) = \mathcal{N}(\boldsymbol{\xi}; \boldsymbol{\mu_\xi}, \boldsymbol{C_\xi})$$

where $\boldsymbol{C_\xi}$ is a covariance matrix. By the way, the mean $\boldsymbol{\mu_\xi}$ is determined by maximizing the quantity $p(\boldsymbol{\xi}|\boldsymbol{o}_{1:K}, \mathcal{A})$.

$$\begin{aligned} \boldsymbol{\mu_\xi} &= \arg\max_{\boldsymbol{\xi}} p(\boldsymbol{\xi}|\boldsymbol{o}_{1:K}, \mathcal{A}) \\ &= \arg\max_{\boldsymbol{\xi}} \frac{p(\boldsymbol{\xi}, \boldsymbol{o}_{1:K}, |\mathcal{A})}{p(\boldsymbol{o}_{1:K}, |\mathcal{A})} \\ &= \arg\max_{\boldsymbol{\xi}} p(\boldsymbol{\xi}, \boldsymbol{o}_{1:K}, \boldsymbol{a}_{1:K}|\mathcal{A}) \end{aligned} \tag{S13}$$

The optimal $q(\boldsymbol{\xi})$ is to maximize negative free energy $\mathcal{F}_{\mathcal{A}}(\boldsymbol{\xi})$

$$\max_{q(\boldsymbol{\xi})} \mathcal{F}_{\mathcal{A}}(\boldsymbol{\xi}) = \max_{q(\boldsymbol{\xi})} \int q(\boldsymbol{\xi}) \ln p(\boldsymbol{o}_{1:K}, \boldsymbol{\xi}|\mathcal{A}) - q(\boldsymbol{\xi}) \ln q(\boldsymbol{\xi}) \mathrm{d}\boldsymbol{\xi}$$

We use the notation $\mathcal{V}(\boldsymbol{\xi})$ to denote the quantity $\ln p(\boldsymbol{o}_{1:K}, \boldsymbol{\xi}|\mathcal{A})$ and then use Taylor's theorem to expand $\mathcal{V}(\boldsymbol{\xi})$ near the point $\boldsymbol{\mu}_{\boldsymbol{\xi}}$

$$\mathcal{V}(\boldsymbol{\xi}) \approx \mathcal{V}(\boldsymbol{\mu}_{\boldsymbol{\xi}}) + \frac{\partial \mathcal{V}(\boldsymbol{\mu}_{\boldsymbol{\xi}})}{\partial \boldsymbol{\xi}}(\boldsymbol{\xi} - \boldsymbol{\mu}_{\boldsymbol{\xi}}) + \frac{1}{2}(\boldsymbol{\xi} - \boldsymbol{\mu}_{\boldsymbol{\xi}})^T \frac{\partial^2 \mathcal{V}(\boldsymbol{\mu}_{\boldsymbol{\xi}})}{\partial \boldsymbol{\xi} \partial \boldsymbol{\xi}^T}(\boldsymbol{\xi} - \boldsymbol{\mu}_{\boldsymbol{\xi}}) \tag{S14}$$

The first item $\int q(\boldsymbol{\xi})\mathcal{V}(\boldsymbol{\xi})\mathrm{d}\boldsymbol{\xi}$ in negative free energy $\mathcal{F}_{\mathcal{A}}(\boldsymbol{\xi})$ can be evaluated by

$$\begin{aligned} \int q(\boldsymbol{\xi})\mathcal{V}(\boldsymbol{\xi})\mathrm{d}\boldsymbol{\xi} \approx& \mathcal{V}(\boldsymbol{\mu}_{\boldsymbol{\xi}}) + \frac{\partial \mathcal{V}(\boldsymbol{\mu}_{\boldsymbol{\xi}})}{\partial \boldsymbol{\xi}} E_{q(\boldsymbol{\xi}|\boldsymbol{\mu}_{\boldsymbol{\xi}}, \boldsymbol{C}_{\boldsymbol{\xi}})}[\boldsymbol{\xi} - \boldsymbol{\mu}_{\boldsymbol{\xi}}] \\ &+ \frac{1}{2} E_{q(\boldsymbol{\xi}|\boldsymbol{\mu}_{\boldsymbol{\xi}}, \boldsymbol{C}_{\boldsymbol{\xi}})}[(\boldsymbol{\xi} - \boldsymbol{\mu}_{\boldsymbol{\xi}})^T \frac{\partial^2 \mathcal{V}(\boldsymbol{\mu}_{\boldsymbol{\xi}})}{\partial \boldsymbol{\xi} \partial \boldsymbol{\xi}^T}(\boldsymbol{\xi} - \boldsymbol{\mu}_{\boldsymbol{\xi}})] \\ =& \mathcal{V}(\boldsymbol{\mu}_{\boldsymbol{\xi}}) + \frac{1}{2} \operatorname{tr}\left(\boldsymbol{C}_{\boldsymbol{\xi}} \frac{\partial^2 \mathcal{V}(\boldsymbol{\mu}_{\boldsymbol{\xi}})}{\partial \boldsymbol{\xi} \partial \boldsymbol{\xi}^T}\right) \end{aligned} \tag{S15}$$

The last item $H_e(\boldsymbol{\xi}) = -\int q(\boldsymbol{\xi}) \ln q(\boldsymbol{\xi}) \mathrm{d}\boldsymbol{\xi}$ is

$$\begin{aligned} H_e(\boldsymbol{\xi}) &= -\int q(\boldsymbol{\xi}) \ln q(\boldsymbol{\xi}) \mathrm{d}\boldsymbol{\xi} \\ &= -E_{q(\boldsymbol{\xi}|\boldsymbol{\mu}_{\boldsymbol{\xi}}, \boldsymbol{C}_{\boldsymbol{\xi}})}\left[\ln q(\boldsymbol{\xi}|\boldsymbol{\mu}_{\boldsymbol{\xi}}, \boldsymbol{C}_{\boldsymbol{\xi}})\right] \\ &= -E_{q(\boldsymbol{\xi}|\boldsymbol{\mu}_{\boldsymbol{\xi}}, \boldsymbol{C}_{\boldsymbol{\xi}})}\left[-\frac{d_{\boldsymbol{\xi}}}{2} \ln 2\pi - \frac{1}{2} \ln \det(\boldsymbol{C}_{\boldsymbol{\xi}}) - \frac{1}{2}(\boldsymbol{\xi} - \boldsymbol{\mu}_{\boldsymbol{\xi}})^T \boldsymbol{C}_{\boldsymbol{\xi}}^{-1}(\boldsymbol{\xi} - \boldsymbol{\mu}_{\boldsymbol{\xi}})\right] \\ &= \frac{d_{\boldsymbol{\xi}}}{2} \ln 2\pi + \frac{1}{2} \ln \det(\boldsymbol{C}_{\boldsymbol{\xi}}) + \frac{1}{2}\operatorname{tr}(I_{d_{\boldsymbol{\xi}}}) \\ &= \frac{d_{\boldsymbol{\xi}}}{2} \ln 2\pi e + \frac{1}{2} \ln \det(\boldsymbol{C}_{\boldsymbol{\xi}}) \end{aligned} \tag{S16}$$

So negative free energy $\mathcal{F}_{\mathcal{A}}(\boldsymbol{\xi})$ can be caculated as

$$\begin{aligned} \mathcal{F}_{\mathcal{A}}(\boldsymbol{\xi}) &= E_{q(\boldsymbol{\xi})}[\mathcal{V}(\boldsymbol{\mu}_{\boldsymbol{\xi}})] + H_e(\boldsymbol{\xi}) \\ &= \mathcal{V}(\boldsymbol{\mu}_{\boldsymbol{\xi}}) + \frac{1}{2}\operatorname{tr}\left(\boldsymbol{C}_{\boldsymbol{\xi}} \frac{\partial^2 \mathcal{V}(\boldsymbol{\mu}_{\boldsymbol{\xi}})}{\partial \boldsymbol{\xi} \partial \boldsymbol{\xi}^T}\right) + \frac{d_{\boldsymbol{\xi}}}{2} \ln 2\pi e + \frac{1}{2} \ln \det(\boldsymbol{C}_{\boldsymbol{\xi}}) \end{aligned} \tag{S17}$$

$\mathcal{F}_{\mathcal{A}}(\boldsymbol{\xi})$ is the scalar valued function of the covariance $\boldsymbol{C}_{\boldsymbol{\xi}}$. $\boldsymbol{C}_{\boldsymbol{\xi}}$ is an optimal point or a stationary point where $\mathcal{F}_{\mathcal{A}}(\boldsymbol{\xi})$ has a maximum value. The partial derivative $\frac{\partial \mathcal{F}_{\mathcal{A}}(\boldsymbol{\xi})}{\partial \boldsymbol{C}_{\boldsymbol{\xi}}}$ is a zero matrix $\boldsymbol{O}$.

$$\begin{aligned} \frac{\partial \mathcal{F}_{\mathcal{A}}(\boldsymbol{\xi})}{\partial \boldsymbol{C}_{\boldsymbol{\xi}}} &= \frac{1}{2}\frac{\partial^2 \mathcal{V}(\boldsymbol{\mu}_{\boldsymbol{\xi}})}{\partial \boldsymbol{\xi} \partial \boldsymbol{\xi}^T} + \frac{1}{2}\boldsymbol{C}_{\boldsymbol{\xi}}^{-1} = \boldsymbol{O} \\ \Longrightarrow \boldsymbol{C}_{\boldsymbol{\xi}} &= -\left(\frac{\partial^2 \mathcal{V}(\boldsymbol{\mu}_{\boldsymbol{\xi}})}{\partial \boldsymbol{\xi} \partial \boldsymbol{\xi}^T}\right)^{-1} \end{aligned} \tag{S18}$$

The maximum value of $\mathcal{F}_{\mathcal{A}}(\boldsymbol{\xi})$ is

$$\mathcal{F}_{\mathcal{A}}(\boldsymbol{\xi}) = \mathcal{V}(\boldsymbol{\mu}_{\boldsymbol{\xi}}) + \frac{d_{\boldsymbol{\xi}}}{2}\ln 2\pi e + \frac{1}{2}\ln \det(\boldsymbol{C}_{\boldsymbol{\xi}}) \tag{S19}$$

4 BAYESIAN MODEL SELECTION

Grounded on probability theory, Bayesian model selection is to evaluate different models based on the observed data, favoring the model with balanced tradeoff between complexity and flexibility. Given a series of input observations $\boldsymbol{o}_{1:K}$, Bayesian model selection is to select the optimal model m^* to best interpret input observations

$$m^* = \arg\max_{m} p(m|\boldsymbol{o}_{1:K}). \tag{S20}$$

Taking two different models m_2, m_1 into account, we can define Bayesian Factor as

$$\begin{aligned} p(m_2|\boldsymbol{o}_{1:K}) &= \frac{p(m_2)p(\boldsymbol{o}_{1:K}|m_2)}{p(\boldsymbol{o}_{1:K})} \\ p(m_1|\boldsymbol{o}_{1:K}) &= \frac{p(m_1)p(\boldsymbol{o}_{1:K}|m_1)}{p(\boldsymbol{o}_{1:K})} \\ \frac{p(m_1|\boldsymbol{o}_{1:K}}{p(m_2|\boldsymbol{o}_{1:K})} &= BF\frac{p(m_1)}{p(m_2)} \\ BF &= \frac{p(\boldsymbol{o}_{1:K}|m_1)}{p(\boldsymbol{o}_{1:K}|m_2)}, \end{aligned} \tag{S21}$$

where $p(m_i)$ is the prior distribution of m_i. Here, we make a general assumption that the prior distribution of a model is a non-informative prior. Under the assumption of non-informative priors, the prior distribution is equivalent to a uniform distribution $\frac{p(m_1)}{p(m_2)} = 1$. Then the ratio of the posterior distributions $\frac{p(m_1|\boldsymbol{o}_{1:K})}{p(m_2|\boldsymbol{o}_{1:K})}$ is simply given by the Bayesian Factor.

Bayesian model selection problem is reduced to selecting a model with maximal model evidence $p(\boldsymbol{o}_{1:K}|m_i)$. In Bayesian learning framework, log-model evidence $\ln p(\boldsymbol{o}_{1:K}|m_i)$ can be approximated by the Bayesian Information Criterion (BIC) Schwarz (1978):

$$\begin{aligned} \ln p(\boldsymbol{o}_{1:K}|m_i) &\approx \ln p(\boldsymbol{o}_{1:K}|\boldsymbol{\mu}_{\boldsymbol{\xi}_i}, m_i) - \frac{d_{\boldsymbol{\xi}_i}}{2}\ln(K) \\ \Rightarrow \ln p(\boldsymbol{o}_{1:K}|m_i) &= \mathcal{F}_{m_i}(\boldsymbol{\mu}_{\boldsymbol{\xi}_i}, \boldsymbol{C}_{\boldsymbol{\xi}_i}) - \frac{d_{\boldsymbol{\xi}_i}}{2}\ln(K) \end{aligned} \tag{S22}$$

where K is the number of the observations in $\boldsymbol{o}_{1:K}$. $d_{\boldsymbol{\xi}_i}$ is the number of free parameters estimated by the model. By computing the negative free energies of two different models $\mathcal{F}_{m_1}(\boldsymbol{\mu}_{\boldsymbol{\xi}_1}, \boldsymbol{C}_{\boldsymbol{\xi}_1}), \mathcal{F}_{m_2}(\boldsymbol{\mu}_{\boldsymbol{\xi}_2}, \boldsymbol{C}_{\boldsymbol{\xi}_2})$, Bayesian Factor is given by

$$\begin{aligned} BF(m_1, m_2) & \frac{p(\boldsymbol{o}_{1:K}|m_1)}{p(\boldsymbol{o}_{1:K}|m_2)} \\ & = \exp(\ln p(\boldsymbol{o}_{1:K}|m_1) - \ln p(\boldsymbol{o}_{1:K}|m_2)) \\ & \approx \exp\left(\mathcal{F}_{m_1}(\boldsymbol{\mu}_{\boldsymbol{\xi}_1}, \boldsymbol{C}_{\boldsymbol{\xi}_1}) - \mathcal{F}_{m_2}(\boldsymbol{\mu}_{\boldsymbol{\xi}_2}, \boldsymbol{C}_{\boldsymbol{\xi}_2}) - \frac{d_{\boldsymbol{\xi}_1} - d_{\boldsymbol{\xi}_2}}{2} \ln(K)\right). \end{aligned} \tag{S23}$$

Bayesian Factor BF	Interpretations
$0 < BF < \frac{1}{100}$	Decisive evidence for m_2
$\frac{1}{100} < BF < \frac{1}{10}$	Strong evidence for m_2
$\frac{1}{10} < BF < \frac{1}{3}$	Moderate evidence for m_2
$\frac{1}{3} < BF < 1$	Weak evidence for m_2
$1 < BF < 3$	Weak evidence for m_1
$3 < BF < 10$	Moderate evidence for m_1
$10 < BF < 100$	Strong evidence for m_1
$BF > 100$	Decisive evidence for m_1

Table S1. Bayes Factors and interpretations

For the ease of using Bayesian Factor, Harold Jeffreys gave a scale for the interpretation of Bayesian Factor (Table.S1) Harold Jeffreys (1961). If $BF > 1$, the model m_1 is more strongly supported by the observed data, and vice versa (if $0 < BF < 1$, the model m_2 is more strongly supported).

REFERENCES

Beal, M. J. (2003). *Variational algorithms for approximate Bayesian inference*. Ph.D. thesis, University College London (UCL)

Harold Jeffreys, S. (1961). *Theory of probability* (New York: Oxford University Press), third edn.

Mathys, C. D., Daunizeau, J., Friston, K. J., and Stephan, K. E. (2011). A bayesian foundation for individual learning under uncertainty. *Frontiers in Human Neuroscience* 5, 39

Schwarz, G. (1978). Estimating the dimension of a model. *The annals of statistics* , 461–464

Zhu, C., Zhou, K., Han, Z., Tang, Y., Tang, F., and Si, B. (2025). General hierarchical Brownian filter in multi-dimensional volatile environments. *submitted*
